# Error-Aware Data Clustering for In-Network Data Reduction in Wireless Sensor Networks

**DOI:** 10.3390/s20041011

**Published:** 2020-02-13

**Authors:** M. K. Alam, Azrina Abd Aziz, S. A. Latif, Azlan Awang

**Affiliations:** 1 Department of Electrical and Electronic Engineering, Universiti Teknologi PETRONAS, Seri Iskandar 32610, Perak, Malaysia; azrina_aaziz@utp.edu.my (A.A.A.); azlan.awang@ieee.org (A.A.); 2 Department of Information Technology, Otago Polytechnic, Dunedin 9016, New Zealand; suhaimi.latif@op.ac.nz

**Keywords:** wireless sensor network, environmental monitoring, time-series clustering, partitional clustering, outlier detection, k-means, k-medoids, in-network data reduction

## Abstract

A wireless sensor network (WSN) deploys hundreds or thousands of nodes that may introduce large-scale data over time. Dealing with such an amount of collected data is a real challenge for energy-constraint sensor nodes. Therefore, numerous research works have been carried out to design efficient data clustering techniques in WSNs to eliminate the amount of redundant data before transmitting them to the sink while preserving their fundamental properties. This paper develops a new error-aware data clustering (EDC) technique at the cluster-heads (CHs) for in-network data reduction. The proposed EDC consists of three adaptive modules that allow users to choose the module that suits their requirements and the quality of the data. The histogram-based data clustering (HDC) module groups temporal correlated data into clusters and eliminates correlated data from each cluster. Recursive outlier detection and smoothing (RODS) with HDC module provides error-aware data clustering, which detects random outliers using temporal correlation of data to maintain data reduction errors within a predefined threshold. Verification of RODS (V-RODS) with HDC module detects not only random outliers but also frequent outliers simultaneously based on both the temporal and spatial correlations of the data. The simulation results show that the proposed EDC is computationally cheap, able to reduce a significant amount of redundant data with minimum error, and provides efficient error-aware data clustering solutions for remote monitoring environmental applications.

## 1. Introduction

Wireless sensor networks (WSNs) have been utilized for various applications such as facility monitoring, environmental monitoring, and military surveillance. Typically, these applications deploy plenty of sensor nodes, which are capable of communicating among themselves and to a base-station or external sink in WSNs [1]. The sensor nodes could be scattered in harsh environments, including battlefields, or deterministically placed at specified locations randomly, and coordinate among themselves to build a communication network. In WSNs, data can be sensed periodically and non-periodically (e.g., event driven). In the event-driven scenario, the sensor nodes sense data only when an event is invoked. For example, the sensor nodes wake up and report the event when they detect the presence of intruders. On the other hand, periodic data monitoring is used for applications where certain conditions or processes need to be monitored continuously, such as temperature or pressure. It monitors a given phenomenon and sends data measurements and notifications to the sink at a regular time interval. This systematic collection of sensed data from periodic applications can be defined as time-series [2]. The features of time-series data are continuous data update, it contains high dimensions and has a large data size.

The transmission of a high volume of time-series data from a source sensor to the sink is inefficient for energy-constraint sensor nodes [3]. In addition, the amount of collected data from a dense sensor network is typically huge for the sink to process. Furthermore, these types of data are often redundant because the environmental data has small variation over time [4], thus the sensor nodes contain similar data in their consecutive readings. Meanwhile, the cluster-head nodes receive redundant readings from neighbour nodes at the same time. As a result, in-network data reduction is needed to aggregate a large number of data into lower-representation. The in-network data reduction refers to the process of eliminating correlated or unnecessary data by either sensor nodes or CHs before transmitting the data to the sink [5]. The main objective of in-network data reduction is to increase the network lifetime and speed-up data analysis in order to make quick decisions. One way to improve the power efficiency is to eliminate the redundant or correlated data before transmitting them to the sink without any large degradation of the accuracy. Unfortunately, the collection of real-world data is usually messy, incomplete, inconsistent, and rarely clean [5]. There can be many reasons for the noisy data, ranging from the environmental obstacle and sensors malfunctioning to human errors during data recording. Thereby, the in-network data reduction is becoming a challenging issue for researchers to handle the noisy sensor data. The data clustering technique is one of the solutions for in-network data reduction. This technique classifies the homogeneous data, removes the redundant data and then forwards the distinct data to the sink.

Data clustering is mostly utilized to reduce correlated data for achieving energy conservation in WSNs [6,7,8,9]. In particular, several data clustering techniques have been explored including principal component analysis based aggregation (PCAg) [10], multiple-PCA [11], candid covariance-free incremental PCA (CCIPCA) [5], data aggregative window function (DAWF) [12], projection basis PCA [13], distributed PCA [14], K-means [15], enhanced K-means [9], K-medoids [16], singular value decomposition (SVD) [17], auto-regressive moving average (ARMA) [18], and least mean square (LMS) [19]. Various applications of these techniques are available in existing literature [20,21,22,23,24,25,26,27,28]. However, current data clustering techniques lead to a myriad of problems including error-control for in-network data reduction, time-intensiveness and complex computation. Apart from this, existing techniques in [9,15,16] are computationally time-intensive due to random initialization process. Indeed, it is challenging to reduce the data, especially with the rapid increase in the amount of data collected by the sensor nodes.

In this paper, a new error-aware data clustering (EDC) technique has been introduced and incorporated at the CHs in WSNs. The EDC is divided into three modules. The first module is histogram-based data clustering (HDC), which aggregates similar data into clusters based on the temporal correlation and provides a systematic data clustering for reducing data redundancy. The second module is recursive outlier detection and smoothing (RODS) with HDC module, which provides the error-bound guaranteed data clustering. This module is capable of detecting random outliers (sudden changes and those that lie outside of the regular data pattern) based on the temporal correlation and replaces them by the normal data. The final module is verification of RODS (V-RODS) with HDC module, which detects not only the random outliers but also frequent outliers (frequent changes and those that lie outside of the regular data pattern of the neighbour nodes) simultaneously. This module considers both the temporal and spatial correlations to detect outliers and replace the outliers with the normal data in order to provide more robust error-aware data clustering. The performance of the proposed EDC has been simulated using real-world sensor data.

The rest of the paper is organized as follows: Section 2 covers various clustering techniques for in-network data reduction in WSNs. Section 3 explains the concepts and definitions related to data clustering. Section 4 introduces a new error-aware data clustering technique for in-network data reduction for WSNs. Section 5 explains the implementation of the proposed technique. Section 6 presents the results and analyses of the proposed technique. Finally, Section 7 concludes the paper.

## 2. Background Works

**Figure 1:**
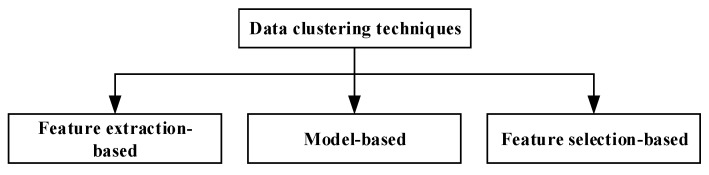
Classification of data clustering techniques for in-network data reduction.

Data clustering has been considered as a useful technique, especially for applications that require scalability to handle a large amount of data collected from the sensor nodes. It also supports data aggregation by removing data redundancy before transmitting them to the sink to gain energy conservation. Recently, in [6,8,29,30], the authors have presented a comprehensive overview of various data clustering techniques for in-network data reduction in periodic WSNs. Generally, these techniques can be classified into three different categories as presented in Figure 1, which are feature extraction-based, model-based and feature selection-based data clustering.

Feature extraction-based technique transforms the original data into a set of data with a reduced number of variables, which contain the most discriminatory information. Usually, this technique is used to reduce data dimensionality by eliminating redundant or irrelevant information. Several feature extraction-based techniques have been explored in [5,13,17,28,31,32,33,34] to handle a large volume of data. These techniques are mostly implemented at the CHs in WSNs, in which the CHs collect data from sensor nodes and transform them into a set of reduced features to remove data redundancy. However, they transform data from the original space into a new space with lower representations, which later cannot be linked to the features in the original space. Therefore, further analysis of the new space is difficult since there is no physical meaning of the transformed features obtained from feature extraction-based techniques. Moreover, feature extraction-based techniques reduce amount of data by selecting the most important feature components from the feature space, thereby the accuracy of these techniques is strongly influenced by the cumulative variance of the selected components. Furthermore, they require extensive computations to transform data to the subspace resulting in an expensive time computation.

Model-based techniques perform data prediction on the collected data and generate a model based on the historical data to reduce the volume of data. These techniques predict the actual reading at the source sensor based on historical recordings and compare predicted reading with the actual reading. If the error between the predicted and actual readings is less than the predefined threshold error, the predicted reading will not be sent to the CHs or sink and vice-versa. Some of the most recent techniques in [18,19,35,36,37,38,39,40,41] are utilized to predict data for in-network data reduction in WSNs, which are simpler, easy to implement and provide acceptable accuracy. Since, the model-based techniques predict data based on historical data, these techniques may not be capable of providing correct prediction when the historical data are noisy and highly inconsistent. Thus, the model-based techniques trade-off the accuracy with data reduction percentage.

Feature selection-based technique is a process to select independent, informative and discriminating features (individual measurable properties or characteristics of a phenomenon being observed [42]) for preprocessing learning algorithms. By using these features, the partitioning data clustering can be performed to eliminate data redundancy as well as improving the learning accuracy, reducing learning time, and simplifying learning results [43]. In machine learning, the feature selection procedure is widely utilized for reducing dimensions of data, especially when dealing with high dimensional space of data [44]. Some of the feature selection-based data clustering techniques are reviewed below, which are mostly related to our proposed technique in this paper.

In [45], the authors propose a distributed K-mean clustering (DKC) procedure for aggregating data at the parent nodes in WSNs. Using DKC, the authors construct a tree-based network to aggregate data from child nodes to parent nodes based on an adaptive weighted allocation. Then, the parent nodes send the reduced data to the sink node. In [46], the authors propose a transmission-efficient technique dedicated to periodic data clustering for underwater WSNs. An enhanced version of K-means algorithm is applied at the CHs to group homogeneous data into clusters for reducing the redundant data. Then, it adopts a one-way ANOVA model with three statistical tests for evaluating the data reduction performance. In [9], an enhanced two-fold EK-means algorithm has been developed to reduce data redundancy, which is the extended version of the previous work in [46]. The first module is executed at the sensor node level, where data are collected in temporal order over time to group similar data into clusters based on the Euclidean distance. The second one is applied at an intermediate node level to aggregate data collected from the neighbour nodes. This module groups spatially correlated data into clusters for reducing data redundancy among neighbour nodes. Furthermore, the work in [47] proposes an algorithm called CluStream, which adopts a k-means procedure to combine similar data in the evolving data streams into clusters.

Apart from these, there are some other similar partitional clustering techniques in the literature, including K-medoids [16], and partitioning around medoids (PAM) [48]. They are introduced to determine the centroids of the clusters in order to reduce redundant data. For example, K-medoids (discrete median ) is a prototype that determines the central value that is an original entity of the input data stream in which the number of data points is odd. If the number of data points is even, it computes the average value of two middle data points of that particular data stream. Another prototype is PAM performing similar to k-mediods. Both prototypes partition data stream into homogeneous groups or clusters based on the sum-of-squared of differences or sum-of-absolute differences. These methods are more robust to noise or outliers as compared to k-means because they reduce the sum-of-pairwise dissimilarities instead of sum-of-squared Euclidean distances. However, its time complexity is higher than the time complexity of k-means in the case of a large data stream.

Furthermore, a hierarchical clustering technique forms a hierarchy or tree of the clusters. There are two types of hierarchical processes: (i) Agglomerative and (ii) divisive. In the agglomerative procedure, a bottom-up strategy is implemented that initially considers data points into a group based on the temporal correlation of the data then the clustering process progresses upwards successively. Similarly, the top-down approach is a divisive process that initially collects all the data points into one group. Then, the algorithm progresses recursively based on similarity measure among the data points to partition the data into the groups. Balanced iterative reducing and clustering utilizing hierarchies (BIRCH) [49] is another popular algorithm for partitioning clustering. This algorithm first summarizes the information of the distribution and performs clustering on the summarized data instead of the original data.

Another technique named histogram-based data clustering has been incorporated in different field of applications [50,51,52] to group similar data into clusters in order to reduce redundant data. The data points of each cluster have been assigned in a systematic way where the random initialization to determine centroids and iteration to converge individual cluster are not required. This technique partitions arranged data into clusters based on the ratio between the range of a data stream and predefined number of clusters. The histogram-based technique is relatively simpler for implementation, and very less execution time is consumed in comparison with other existing techniques [9,16,46].

However, the main constraint of the partitioning data clustering is the determination of the number of clusters without any prior knowledge about the data. Thereby, the performance of many existing partitioning data clustering techniques are highly affected by the quality of data. The inconsistent, irregular and noisy, data can change the range of a data stream and influence the clustering accuracy. Most of the existing techniques only focus on clustering data that are falling within the user-defined threshold value instead of repairing the data, which are outbound of the predefined threshold. Moreover, the current partitional clustering algorithms [9,16,46] may not be suitable for in-network data reduction due to the random initialization in determining centroids of the clusters. Furthermore, the formation of hierarchical tree structure in the hierarchical clustering algorithms is computationally expensive and may not be appropriate for a large dataset. Moreover, none of the current data clustering techniques focuses on the improvement of data reduction rate and data reduction accuracy simultaneously. Therefore, this paper develops the partitioning data clustering technique and considers noisy data in order to achieve maximum data reduction and minimum reduction errors.

## 3. Concepts and Definitions

The basic concepts and definitions utilized in this work are provided in this section. The key notations used in this paper are presented in Table 1.

**Definition** **1.** ***(A Time-Series)***: *A time-series is a set of numerical values that indicates the temporal characteristics of sensor node i (i=1,2,3,⋯,m) at any specific time point (t) of the total lifetime T [53] as given in Equation (1):*

(1) $$X_{i}={\left[x_{i,1}\left(t\right),x_{i,2}\left(t\right),x_{i,3}\left(t\right)\cdots x_{i,n}\left(t\right)\right]}^{T},$$

*where n is the total number of time points.*

**Definition** **2.** ***(Range)***: *The range $\Delta_{d}$ is a numerical indication of the span of a set of data in a distribution or data stream where subscript d indicates “data stream". To specify the range, we simply identify the min and max values of that particular set of data in a data stream and then subtract the min from max. The range of a set of data can be formulated as in Equation (2):*

(2) $$\Delta_{d}=\left(\max\left(X_{i}\right)-\min\left(X_{i}\right)\right).$$

**Definition** **3.** ***(Mean)***: *Mean is a central value of a collection of data in a distribution [54]. For example, $X_{i}$ is a collection of numerical observations of distribution, then the mean μ of this particular distribution can be expressed as in Equation (3):*

(3) $$\mu =\frac{1}{n}\left(\sum_{i=1}^{n}X_{i}\right),$$

*where $X_{i}\in X$.*

**Definition** **4.** ***(Median)***: *The median is the middle or central value of a collection of arranged data [54]. If the number of observations of a collection of data is odd, then the median value is set as the middle data point in the collection of arranged observations. Otherwise, the mean of two values is computed and set as the median. The median M can be formulated as in Equation (4):*

(4) $$M=\left\{\begin{array}{c} {\left(\frac{n+1}{2}\right)}^{th}\begin{array}{c} \mathrm{element},\;\;\;\;\;\;\;\mathrm{Odd} \end{array}\\ \frac{1}{2}\left({\left(\frac{n}{2}\right)}^{th}+{\left(\frac{n}{2}+1\right)}^{th}\right)\mathrm{element},\;\mathrm{Even} \end{array}\right.,$$

*where n is the number of possible arranged elements or values of a given distribution.*

**Definition** **5.** ***(Standard Deviation)***: *The standard deviation is a measure that is used to quantify the amount of variation or dispersion of a set of data values [54]. It is also useful in comparing sets of data that may have the same mean but a different range. For example, the mean of the following two sets is the same: 15, 15, 15, 14, 16 and 2, 7, 14, 22, 30. However, the possible values of the first set are mostly redundant and less spread out compared to the second set. The standard deviation can be defined by σ and computed as in Equation (5):*

(5) $$\sigma =\sqrt{\frac{1}{n}\left(\sum_{i=1}^{n}{\left(X_{i}-\mu \right)}^{2}\right)}.$$

**Definition** **6.** ***(Data Redundancy)***: *Redundancy is the provision of additional or duplicate resources, which can produce similar results. Usually, improving the consistency of observations provided by WSNs is to increase the redundancy, by either obtaining observations for a specific location from multiple neighbouring sensor nodes in a particular time (spatial redundancy), or by obtaining several observations for a specific location from the same sensor node over time (temporal redundancy) [55].*

**Definition** **7.** ***(Spatial Data Clustering)***: *A set of data streams D is collected from the m sensor nodes hence, $D=\left\{X_{1},X_{2},X_{3},\cdots \cdots ,X_{m}\right\}$ where $X_{i}$ (i=1,2,3,…,m). The unsupervised clustering process of D into $C=\left\{C_{1},C_{2},C_{3},\cdots \cdots ,C_{p}\right\}$, occurs such that homogeneous neighbour sensor nodes are grouped together based on the application requirements, a group that is called spatial data clustering. $C_{j}$ is then called a cluster, where $C={⋃}_{j=1}^{p}C_{j}$ and $C_{j}\cap C_{f}=\emptyset$ for 1≤j≠f≤p.*

**Definition** **8.** ***(Temporal Data Clustering)***: *A temporal data cluster $SC_{j}$ is a set of individual time-series data that are similar in time collected from a sensor node. Time-series data are considered to a new cluster based on their contiguous non-overlapping interval, which is a constant partition of the range of a set of data in a distribution. Hence, $B=\left\{SC_{1},SC_{2},SC_{3},\cdots \cdots ,SC_{k}\right\}$ is the set of clusters, where $B={⋃}_{j=1}^{k}SC_{j}$ and $\begin{array}{c} \begin{array}{c} SC_{j} \end{array} \end{array}\cap SC_{f}=\emptyset$ for $\begin{array}{c} 1\leq j\neq f\leq k \end{array}$ and k<p<<m.*

## 4. The Proposed Error-Aware Data Clustering (EDC) Technique

**Figure 2:**
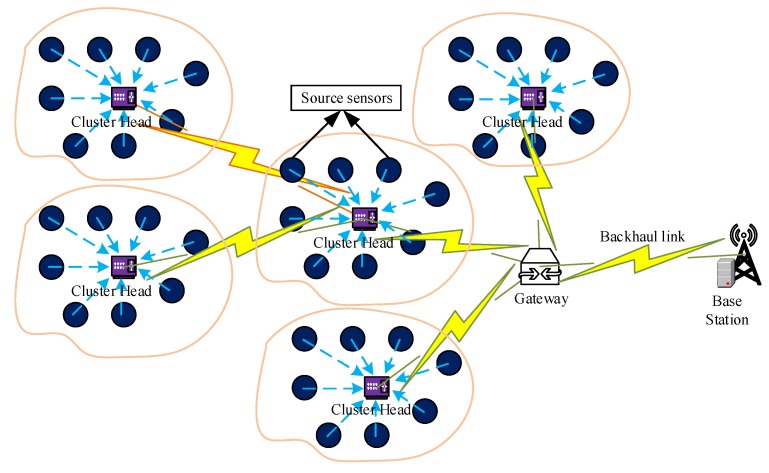
A scenario of a periodic wireless sensor network (WSN) for a long-term monitoring application.

A new error-bound guaranteed data clustering technique has been proposed to reduce the data redundancy by keeping the data reduction error within the user-defined threshold. This technique is mainly divided into three adaptive modules, namely histogram-based data clustering (HDC), recursive outlier detection and smoothing (RODS) with HDC, and verification of RODS (V-RODS) with HDC. The users are allowed to choose any of these modules for performing data clustering based on their requirements and quality of data. The proposed EDC technique has been implemented at each cluster-head (CH) of WSNs and data clustering is performed simultaneously to achieve in-network data reduction effectively. An example of a WSN scenario has been presented in Figure 2 where the source sensors, CHs, gateway, base station and other communication links are highlighted.

The sensor nodes with limited computational capacity and power supply are designed to be responsible for sampling the application surroundings and transmitting the sampled sensor data to their CH. However, we assume that CH is equipped with the powerful hardware capabilities to aggregate the data from multiple sensor nodes. In WSNs, each CH in a cluster collect the sensor readings from sensor nodes in a constant time interval τ. The collected readings can be arranged in the form of a data matrix for every constant time interval. The data matrix ${\overset{⌢}{X}}_{m\times n}$ can be expressed mathematically as in Equation (6):

(6) $${\overset{⌢}{X}}_{m\times n}=\left[\begin{array}{c} {\overset{⌢}{x}}_{1,1}\\ {\overset{⌢}{x}}_{2,1}\\ \vdots \\ {\overset{⌢}{x}}_{m,1} \end{array}\begin{array}{c} {\overset{⌢}{x}}_{1,2}\\ {\overset{⌢}{x}}_{2,2}\\ \vdots \\ {\overset{⌢}{x}}_{m,2} \end{array}\begin{array}{c} \cdots \\ \cdots \\ \ddots \\ \cdots \end{array}\begin{array}{c} {\overset{⌢}{x}}_{1,n}\\ {\overset{⌢}{x}}_{2,n}\\ \vdots \\ {\overset{⌢}{x}}_{m,n} \end{array}\right],$$

where *m* and *n* are the total number of sensor nodes of a cluster and the total number of readings, respectively.

During the collection of data, missing values can occur in periodic WSNs due to a variety of causes such as malfunctioning of sensor nodes, communication failure, interference, and unsynchronized sampling of the sensors. The missing values in a dataset could be appeared as: ‘NaN’, ‘Na’, ‘N123’, ‘empty string’ and ‘in finitive values’. If there is any missing data in ${\overset{⌢}{X}}_{m\times n}$, then it will be detected and replaced by the computed median $M_{D}$ (as in Equation (4)) value of the particular data matrix ${\overset{⌢}{X}}_{m\times n}$ in order to maintain the full sample size for further analysis, where subscript *D* indicates "data matrix". Each collected reading of the sensor node *i* at time instant $t\left(t=1,2,3,\cdots ,T\right)$ in a continuous-time series data stream of a data matrix ${\overset{⌢}{X}}_{m\times n}$, is expressed as in Equation (7):

(7) $$X_{i}\left[t\right]=X_{i}^{*}\left[t\right]+\omega_{i}\left[t\right],$$

where *i* is the spatial location identifier and *t* is the discrete-time identifier.

Consequently, all sensor nodes are assumed to be synchronized according to the discrete-time model [56] where *m* is the number of spatial locations, which is equal to the number of sensor nodes (i.e., each location is covered by a single sensor). $X_{i}^{*}\left[t\right]$ is the noiseless physical phenomenon value (e.g., a temperature reading value), and the noise values ${\left\{\omega_{i}{\left[t\right]}^{˜}N\left(0,\sigma_{\omega }^{2}\right)\right\}}_{i=1}^{m}$ are independent and identically distributed (i.i.d) random Gaussian variables with zero mean and variance $\sigma_{\omega }^{2}$ that depend on the hardware accuracy [56]. Furthermore, we assume that $\varphi_{1}<X_{i}\left[t\right]<\varphi_{2}$ as the dynamic sensing range of the sensor nodes with $\varphi_{1}$ and $\varphi_{2}$ as constant values that are usually given in hardware datasheets. Thereby, any sample value that falls outside this sensing range is considered as an invalid reading (e.g., generated by a defective sensor). For example, the temperature monitoring sensor (model Mica2Dot) [57] measures the temperature in the range of −10 to 70 °C. Therefore, any reading beyond this range is considered as invalid data and have been replaced with the median $M_{i}$ value of the particular data stream. The mean value is highly sensitive to noise [58], thereby median value has been selected instead. The further details of the proposed technique are explained in the following subsections.

### 4.1. Histrogram-Based Data Clustering (HDC)

The HDC is a partitional-based data clustering technique broadly used in data mining to discretize continuous time-series variables [50,52,59]. Another work in [51], initially introduced the concept of HDC as a sampling algorithm for periodic WSNs. This algorithm partitions similar sensor data into clusters to minimize data redundancy by sampling random or central elements of each cluster. In this paper, the HDC is implemented in low-power periodic WSNs to monitor long-term environmental applications and tested with the real-world sensor data. The main objective of the HDC is to provide in-network data reduction facility with limited discretization error. The HDC technique is listed in Algorithm 1, which can be divided into three major phases: (i) Initialization determines the number of clusters and the range of a data stream, computes the constant cluster interval and updates the all clusters intervals recursively according to the lines 1–3. (ii) Data clustering phase assigns data elements from a data stream into the clusters based on the given intervals and reforms the empty clusters if any as shown in lines 5–7. (iii) Data centring and aggregation determines the central value of each cluster using different prototypes (e.g., mean, medoids) for representing that particular cluster. Then, it computes the mean deviation error of all clusters and aggregates the discretized data stream according to the lines 8–10. The detail procedures are explained mathematically in the following sub-sections.

**Algorithm 1:** Histogram-based data clustering and aggregation

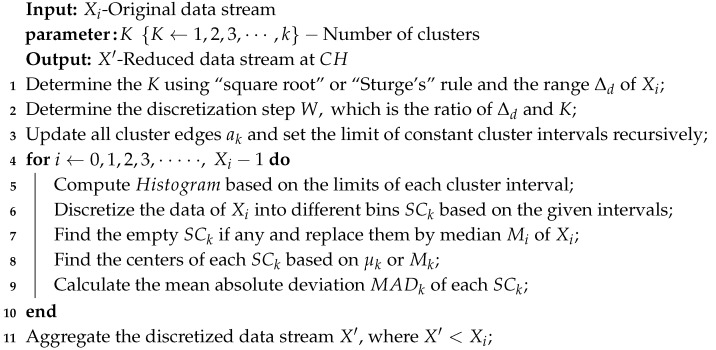



#### 4.1.1. Initialization Phase

In this phase, parameters in HDC are initialized and computed. The number of bins or clusters K (K=1,2,3,⋯,k, where *k* is defined as an individual cluster) is an important parameter that is defined initially for HDC using two different rules, namely the square-root rule [54] and Sturge’s rule [60]. These rules are mostly used for normal or Gaussian distribution. Using these rules, the optimum number of clusters is defined by $K_{sqrt}$ and $K_{Sturge^{\prime }s}$, respectively, and its constant interval-width *W* ($W=w_{1},w_{2},\cdots \cdots ,w_{n}$) are calculated as in Equations (8) and (9):

(8) $$K_{sqrt}=\left(\left\lceil \sqrt{\left|X_{i}\right|}\right\rceil \right)\;\mathrm{and}\;w_{1}=\left(\frac{\max\;X_{i}-\min\;X_{i}}{K_{sqrt}}\right),$$

(9) $$K_{Sturge^{\prime }s}=\left(\left\lceil 1+3.322\left(\log_{2}\left|X_{i}\right|\right)\right\rceil \right)\;\mathrm{and}\;w_{2}=\left(\frac{\max\;X_{i}-\min\;X_{i}}{K_{Sturge^{\prime }s}}\right).$$

#### 4.1.2. Data Clustering Phase

The sequence of edges of the clusters is calculated according to the arithmetic or linear sequence. In a sequence of computed edges, each new term is calculated by adding a constant interval-width *W* to the previous term. For example, the difference between any two adjacent edges is *W*. The recursive definition is therefore as in Equation (10):

(10) $$a_{k}=a_{k-1}+W,\;a_{1}=a_{0}+W,$$

where the term $a_{k}=a_{1},a_{2},a_{3},\cdots a_{k}$ and $a_{0}=\min\left(X_{i}\right)$ is defined as the lower bound of edges as well as the clusters K constant intervals $\left(a_{0},a_{1}\right],\left(a_{1},a_{2}\right],\left(a_{2},a_{3}\right],\cdots ,\left(a_{k-1},a_{k}\right]$. The data elements that fall between the lower-bound edge and upper-bound edge (e.g., $a_{0}$ and $a_{1}$) of a cluster, then they are considered for that particular cluster. If there is no element (i.e., empty cluster) in any cluster $SC_{k}$, then that empty cluster will be detected and replaced with the computed median $M_{k}$ value of the particular data stream $X_{i}$. This replacement is done in order to maintain the equal length of the aggregated data with respect to the aggregated data from other data streams.

#### 4.1.3. Centering and Aggregation Phase

In this phase, we determine the central value of each cluster using two different prototypes including averaging and medoids as well as computing the absolute deviation error of each data point of each cluster. Here, we consider two clusters of datasets as an example to calculate central values and absolute deviation errors. The clusters of datasets can be expressed as $SC_{1}=\left\{d_{0},d_{1},d_{2},\cdots ,d_{L}\right\}$ and $SC_{2}=\left\{d_{L+1},d_{L+2},d_{L+3},\cdots ,d_{R-1}\right\}$, whereas the central values of the clusters have been computed based on Equations (3) and (4).

The absolute deviation error (ADE) is a distance between a particular data point and the central value, which can be calculated for each data point of each cluster as in Equation (11):

(11) $$ADE_{i}=\left(|d_{i}-\mu_{k}|\right)\;\mathrm{or}\;ADE_{i}=\left(|d_{i}-M_{k}|\right).$$

The mean absolute deviation of an individual cluster, as well as all clusters of a data stream, are computed as in Equations (12) and (13), respectively:

(12) $$MAD_{k}=\frac{1}{L}\sum_{i=0}^{L}ADE_{i},$$

(13) $$MAD_{B}=\frac{1}{k}\sum_{i=0}^{k}\left(|MAD_{k}-\mu_{K}|\right).$$

**Figure 3:**
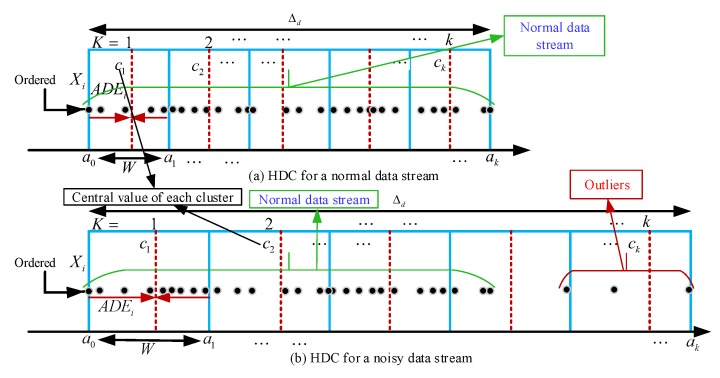
A graphical representation of the histogram-based data clustering (HDC) technique. (**a**) The HDC performs clustering for a “normal” data stream; (**b**) The HDC performs clustering for a “noisy” data stream.

The main advantage of the HDC technique is its ability to perform data clustering in a systematic way. There is no random initialization to determine the initial centroids for the number of clusters of a dataset as like existing data clustering techniques in [9,46]. However, in the HDC as well as other partitioning data clustering techniques [9,46], the number of clusters is required to be predefined without any prior knowledge about the datasets. Thus, the main constraint of these techniques is to decide an optimum number of clusters based on the variation of the data. Even though the HDC works well in the case of “normal” data streams (the consecutive data proximity are very high in temporal order) as shown in Figure 3a, the issue emerges at the presence of “outliers” (the sudden change between the consecutive readings called noisy data streams) as presented in Figure 3b.

In WSNs, outliers can be defined as the sensor readings that significantly deviate from the normal pattern of the sensed dataset [61]. The outliers can be caused by the sensor malfunction, faulty sensor nodes, noise, and error readings. Two types of outliers might be generated, namely randomly generated outliers and frequently generated outliers. Generally, outliers can dramatically change the normal data pattern and affect the overall data analysis [62]. For example, in Figure 3b, when outliers occur in a data stream $X_{i}$, the range $\Delta_{d}$ of $X_{i}$, increases significantly, as well as cluster width *W*. As a result, the data proximity may be reduced, the central values of the clusters may be shifted, and the deviation error may be increased. Therefore, in this paper, the RODS technique has been introduced with HDC to handle outliers to facilitate the process of determining the number of clusters.

### 4.2. Recursive Outlier Detection and Smoothing (RODS) with HDC

The main purpose of introducing the RODS with HDC is to consider noisy data during clustering in order to achieve error-aware data reduction. The proposed RODS processes the data stream according to Algorithm 2 for detecting and smoothing the outlier data points that exist in the data stream recursively. The RODS can be divided into two major phases, including initialization and recursion. In the initialization phase, the noisy data stream is detected and re-scaled by the standard score (z_score) method. Then, the standardized scores are initialized to determine outlier data points according to the lines 1–4. In recursion phase, if the outlier data point is detected, then it is replaced with the median value of that particular standardized scores. Finally, the updated standardized scores are reconstructed and the process is repeated recursively until the user requirements are fulfilled as shown in lines 6–11. Otherwise, the Algorithm 1 is run to perform data clustering and aggregation for reducing the redundancy of a given data stream. The mathematical explanations of the RODS and its different phases are discussed below: 

**Algorithm 2:** Recursive outlier detection and smoothing (RODS) with HDC

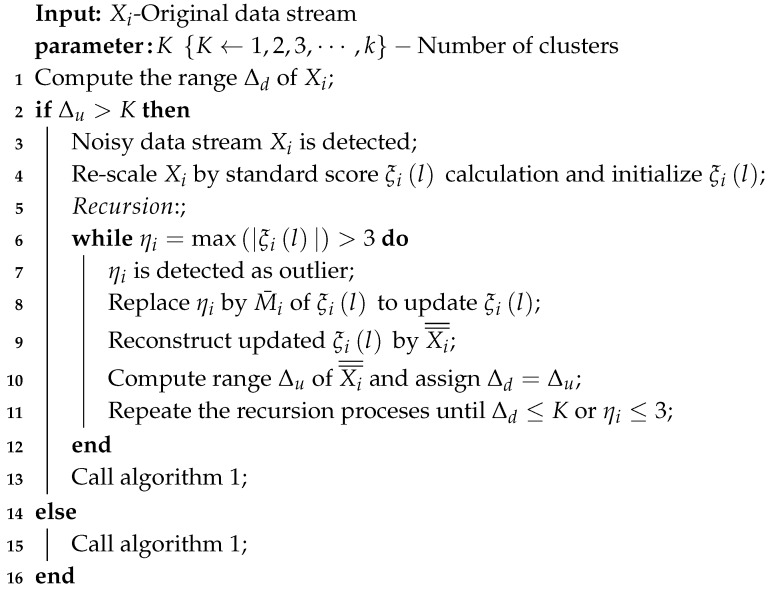



In the RODS technique, a data stream $X_{i}$ is detected as noisy data based on the comparison between the computed range $\Delta_{d}$ of a particular data stream as in Equation (2) and the predefined number of clusters K. If $\Delta_{d}$ of $X_{i}$ is higher than K, then the $X_{i}$ is considered as “noisy”, otherwise it is detected as “normal” as in Equation (14):

(14) $$\left\{\begin{array}{l} \mathrm{if}\;\begin{array}{c} \Delta_{d}>K\;\mathrm{Noisy}\mathrm{data}\mathrm{stream} \end{array}\\ \mathrm{if}\;\begin{array}{c} \Delta_{d} \end{array}\leq K\;\mathrm{Normal}\mathrm{data}\mathrm{stream} \end{array}\right..$$

#### 4.2.1. Initialization Phase

The raw sensor data stream $X_{i}$, which has been detected as “noisy”, is re-scaled based on the standard score method where the mean is 0, and the standard deviation is 1 (i.e., normal distribution). The standardization of a data stream is carried out to avoid influence of different raw data scales as in Equation (15):

(15) $$\xi_{i}\left(l\right)=\frac{X_{i}\left(l\right)-\mu_{i}}{\sigma_{i}},l=1,2,3,\cdots ,n,$$

where $\mu_{i}$ and $\sigma_{i}$ are the mean and standard deviation of $X_{i}$.

#### 4.2.2. Recursion Phase

In this phase, the maximum absolute standard score $\eta_{i}$ in $\xi_{i}\left(l\right)$ is determined by $\eta_{i}=\max\left(|\xi_{i}\left(l\right)|\right)$. Based on the empirical rule, if the maximum absolute score $\eta_{i}>3$ then it is considered as outlier. According to this rule, it is proven that almost all of the data (99.7%) should fall within three standard deviations from the mean [63]. Afterwards, the recursion processes are performed as follows: (i) The detected outlier $\eta_{i}$ is replaced with the computed median ${\bar{M}}_{i}$ of $\xi_{i}\left(l\right)$ to update the standardized data stream $\xi_{i}\left(l\right)$; (ii) the updated $\xi_{i}\left(l\right)$ is de-standardized by $\bar{\bar{X_{i}}}=\left(\xi_{i}\left(l\right)\times \sigma_{i}\right)+\mu_{i}$; (iii) the range $\Delta_{u}$ of the updated de-standardized data stream $\bar{\bar{X_{i}}}$ where subscript *u* indicates “updated data stream” is computed; (iv) it is assigned to $\Delta_{d}$ ($\Delta_{d}=\Delta_{u}$); (v) the updated $\Delta_{d}$ is compared with K. These processes are repeated recursively until the $\Delta_{d}$ value is less than K or $\eta_{i}\leq 3$. If there is no outlier in the data stream then the HDC technique is used to perform data clustering and aggregation.

**Figure 4:**
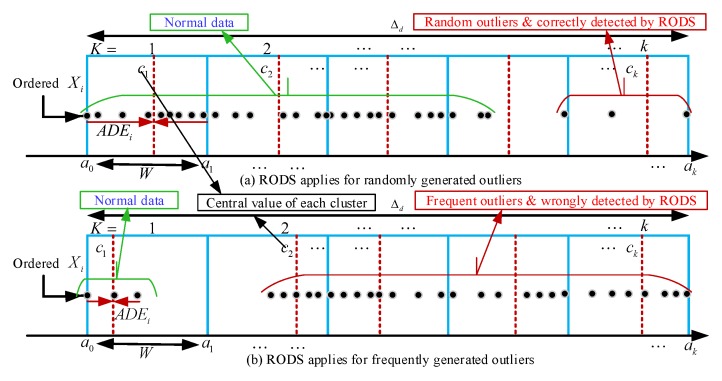
Error-aware data clustering technique by considering noisy data stream. (**a**) Recursive outlier detection and smoothing (RODS) applies for detecting random outliers; (**b**) RODS applies for detecting frequent outliers.

The RODS with HDC provides an error-bound guaranteed data clustering facility to reduce in-network data redundancy for the periodical sensing data in WSNs. However, the RODS detects outliers only by examining an individual data stream of a sensor node and performed outlier detection based on temporal correlation [58]. Temporal correlation refers to the proximity of data collected over time from a specific sensor node. The RODS therefore can only detect the random outliers but not the frequently generated outliers because it has no knowledge of the spatial correlation of the data stream. Spatial correlation refers to the similarity of data among different sensor nodes at a specific time point or period. Besides, the RODS detects outliers based on the standard score method, which relies on the mean and standard deviation of a data stream to measure central tendency and dispersion. This method may not be robust due to the mean value being highly affected by the frequently generated outliers. Figure 4 presents two different types of outliers in a data stream.

Figure 4a shows a noisy data stream where the random outliers are present in fewer numbers compared to normal data points. The proposed RODS detects random outliers correctly based on the temporal correlation among data in a data stream. On the other hand, Figure 4b presents the noisy data stream where the frequent outliers exist in larger numbers in contrast to normal data points. In this case, the RODS detects the outliers wrongly due to the temporal correlation-based detection. Therefore, the V-RODS has been proposed in this paper to detect both the random and frequent outliers effectively. This technique utilizes both temporal and spatial correlations of the data stream for outlier detection.

### 4.3. Verification of Recursive Outlier Detection and Smoothing (V-RODS) with HDC

The V-RODS uses a modified standard score method where mean value is replaced with the median value of an outlier in data stream. Moreover, it considers the data of neighbouring nodes in a CH and utilizes the spatial correlation of the sensing data streams instead of only the temporal correlation of that particular data stream. Hence, the V-RODS in Algorithm 3 is able to detect and replace both types of outliers of a data stream more precisely. After detecting noisy data stream based on the Equation in (14), the V-RODS is initiated to detect and repair both types of outliers in the data stream. This technique is separated into two phases, including initialization and recursion. In the initialization phase, the noisy data stream is detected and re-scaled using the modified standard score method. Then, the V-RODS is utilized for detecting outlier data points from the standardized scores according to the lines 1–4. In the recursion phase, the outlier data point is detected from the standardized scores based on the temporal correlation and then de-standardized the detected outlier. Later on, the de-standardized data point is compared to the median value of the neighbouring data streams for verification, to check whether it is false detection or not. Here, the neighbouring data streams have similar length and same period as to the detected noisy data stream. When outlier is verified as a false detection, then we consider that outlier as frequent outlier and the data stream is marked as invalid. After that, the modified standardized scores are de-standardized for replacing the actual outliers with the valid data recursively according to the lines 8–15. Otherwise, Algorithm 2 is executed to handle the detected outlier from the data stream. Afterwards, the recursion process is repeated until the user requirements meet lines 6–17. Finally, the HDC is executed to perform data clustering for the noise-free data stream in order to achieve error-bound guaranteed data reduction. The mathematical explanations are explained in detail below:

**Algorithm 3:** Verification of RODS (V-RODS) with HDC

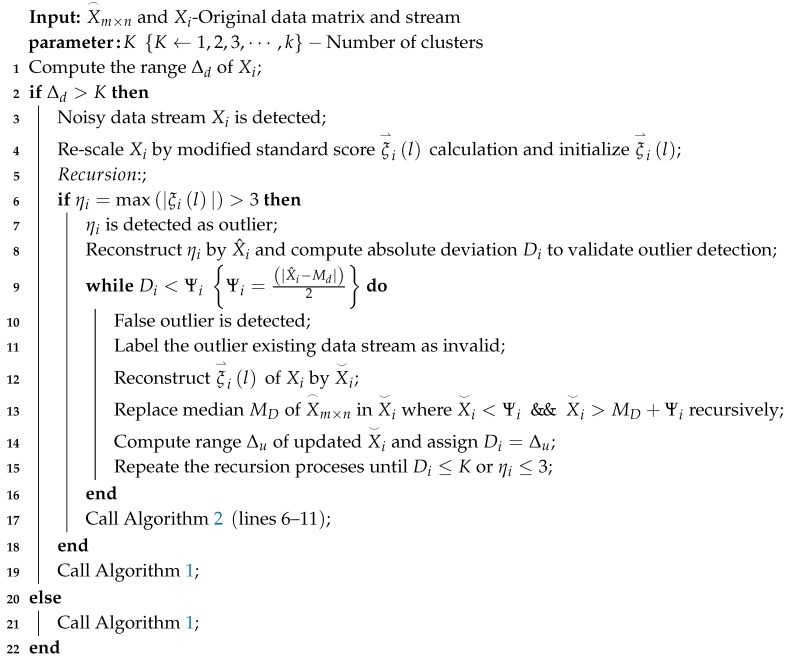



#### 4.3.1. Initialization Phase

In this phase, initially outliers existing data stream $X_{i}$ is given, and then the median value $M_{i}$ of the $X_{i}$ as well as the median value $M_{D}$ of the particular data matrix ${\overset{⌢}{X}}_{m\times n}$ where i=1,2,3,⋯,m is calculated. After that, the modified standard score of the $X_{i}$ is calculated using the computed $M_{i}$ as in Equation (16):

(16) $${\overset{⇀}{\xi }}_{i}\left(l\right)=\frac{X_{i}\left(l\right)-M_{i}}{\sigma_{i}},l=1,2,3,\cdots ,n.$$

Then, an outlier of the $X_{i}$ is detected by $\eta_{i}=\max\left(|{\overset{⇀}{\xi }}_{i}\left(l\right)|\right)$ while $\eta_{i}>3$. To validate the correct outlier, the detected outlier is de-standardized based on Equation (17):

(17) $${\hat{X}}_{i}=\left(\eta_{i}\times \sigma_{i}\right)+M_{i},$$

where ${\hat{X}}_{i}$ is a de-standardized outlier data point.

The absolute deviation $D_{i}$ between the de-standardized outlier ${\hat{X}}_{i}$ and the computed median value $M_{D}$ is calculated by $D_{i}=\left(|{\hat{X}}_{i}-M_{D}|\right)$ to examine the data proximity between the detected outlier ${\hat{X}}_{i}$ and the central value of the particular data matrix. The final decision on the correct outlier detection is examined in the recursion phase.

#### 4.3.2. Recursion Phase

In the recursion phase, the absolute deviation $D_{i}$ is compared to the user-defined threshold value $\Psi_{i}$, which is calculated by $\Psi_{i}=\frac{\left(|{\hat{X}}_{i}-M_{d}|\right)}{2}$ where $M_{d}$ is the median value of the particular data stream $X_{i}$. Now, it can be examined that the former detected outlier is either actual or false outlier based on Equation (18):

(18) $$\left\{\begin{array}{l} \mathrm{if}\;\begin{array}{c} D_{i}<\Psi_{i}\;\mathrm{False}\mathrm{outlier} \end{array}\\ \mathrm{if}\;\begin{array}{c} D_{i} \end{array}\geq \Psi_{i}\;\mathrm{Actual}\mathrm{outlier} \end{array}\right..$$

If the former outlier detection is examined as false detection, then we label the false outlier detected data stream $X_{i}$ as invalid. The invalid data stream can be excluded or repaired for further analysis, depending on the basis of the availability of computational resources. In this paper, the invalid data stream has been repaired by reconstructing the standardized scores ${\overset{⇀}{\xi }}_{i}\left(l\right)$ of $X_{i}$ using Equation (19):

(19) $${\overset{⌣}{X}}_{i}=\left({\overset{⇀}{\xi }}_{i}\left(l\right)\times \sigma_{i}\right)+M_{i},$$

where ${\overset{⌣}{X}}_{i}$ is the de-standardized data stream of the modified standardized scores ${\overset{⇀}{\xi }}_{i}\left(l\right)$.

After de-standardization, the data points of the de-standardized data stream are replaced with the $M_{D}$ where ${\overset{⌣}{X}}_{i}<\Psi_{i}\;\&\&\;{\overset{⌣}{X}}_{i}>M_{D}+\Psi_{i}$ recursively. However, if the former outlier detection is examined as actual outlier, then the same procedures of the RODS in Section 4.2.2 are repeated.

## 5. Implementation of the Proposed EDC

**Figure 5:**
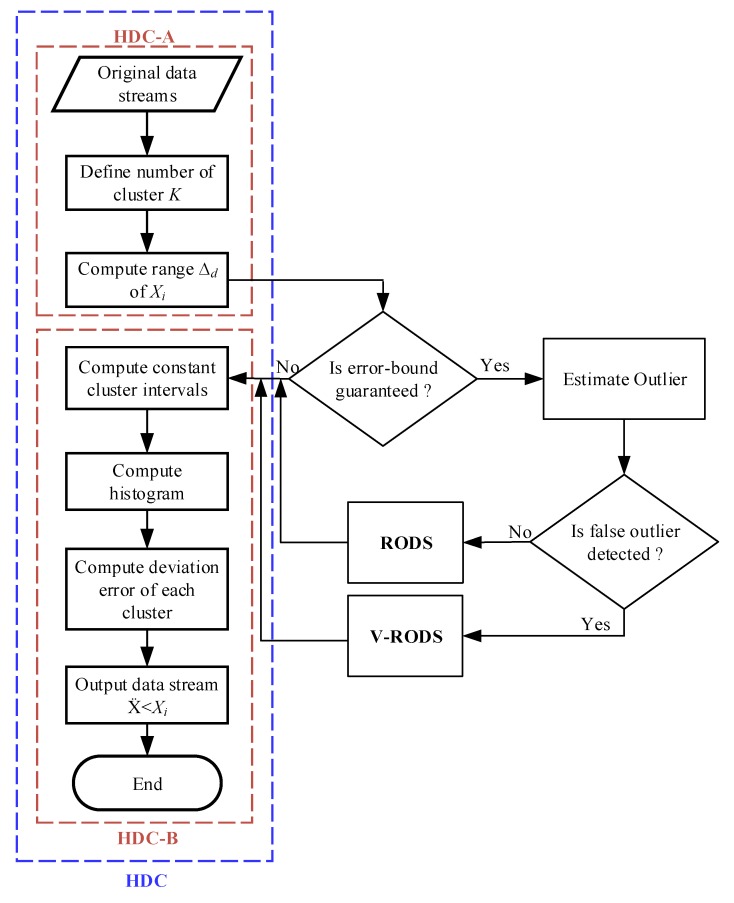
Execution flowchart of the proposed error-aware data clustering (EDC) technique.

**Figure 6:**
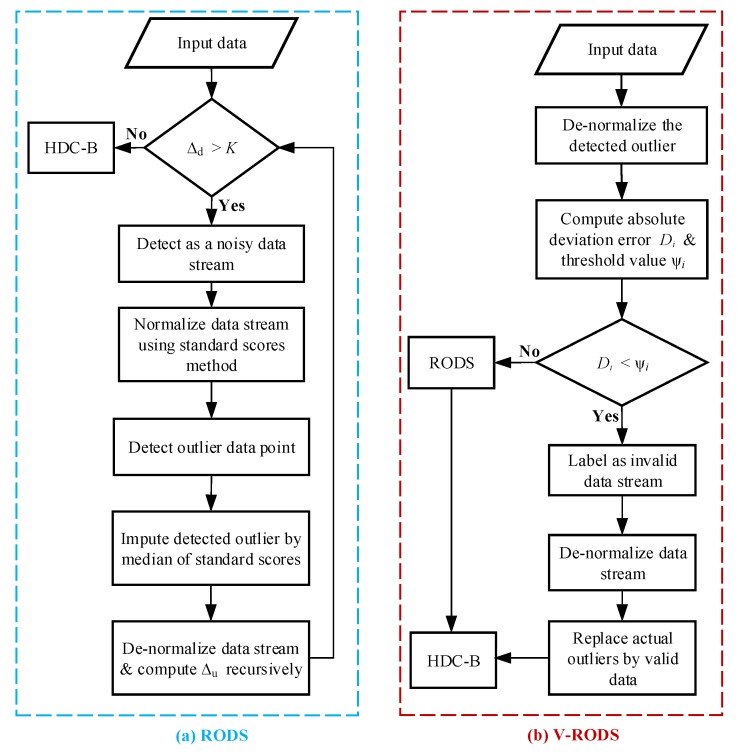
Execution flowcharts of the two modules of the EDC technique. (**a**) RODS with HDC; (**b**) V-RODS with HDC.

The proposed EDC has been implemented at CH to reduce the temporal correlated data points that are generated from the connected sensor nodes in each cluster. For example, $X_{1\times n}\left[T\right]$ is the collected data points from a sensor node at the CH within a certain period where *n* is the number of observations of the stated phenomenon. The EDC is executed by the multiple CHs running simultaneously where all CHs are connected with a sink node, either in direct or multi-hop connection. For simplicity, each CH in each cluster is assumed to have equal number of sensor nodes connected to it. Figure 5 presents the flowchart of EDC technique, which provides the histogram-based data clustering and error-bound guaranteed data reduction. As mentioned earlier, the EDC consists of three main modules, the flow of operations for first HDC module is highlighted in Figure 5 and the rest of two modules are presented in Figure 6. The HDC module groups similar data into clusters based on temporal correlation and the data of each cluster are represented by the central value of that particular cluster for in-network data reduction. Thus, the overall changes in environment variables can be monitored efficiently from the remote server by transmitting the reduced set of data instead of a large data stream. However, the closeness between the reduced set of data and original data stream is measured by the deviations between the central value of each cluster and the all data points of that cluster. If the average deviation is too high, then the changes in the trend of the variables might be significant and abnormal. Otherwise, the variables follow the normal trend even after significant data reduction. Afterwards, the RODS module in Figure 6a is implemented to maintain the deviation error within the user-defined threshold before clustering the data. This module detects the random outliers (noisy data) based on the temporal similarity of the input data stream because the presence of these outliers may introduce high deviation error during data clustering. Then, the detected outliers are replaced with the normal data recursively until the average deviation error meets the predefined threshold. Finally, the detected outliers are verified by the V-RODS module, which is presented in Figure 6b. The V-RODS module utilizes both the temporal and spatial correlations to handle random and frequent outliers. The frequent outliers are classified according to the neighbour knowledge of a CH and then they are replaced by the valid data points.

### 5.1. Dataset

The Intel Berkeley Research Lab (IBRL) dataset [57] has been utilized to evaluate the performance of our proposed EDC technique. This dataset was commonly used to evaluate the performance of many existing data clustering techniques in order to gain in-network data reduction in WSNs [5,13,15,22,23,32,64]. The IBRL dataset was collected using a WSN deployed in Intel Laboratory at the University of Berkeley. The WSN consists of 54 Mica2Dot sensor nodes that were deployed in a duration of 38 days from 28 February to 5 April in 2004. There were four types of measurements (temperature, humidity, light, and voltage), which were recorded in every 31 s interval using this network. The IBRL dataset is considered as a type of static dataset because it is deployed inside the lab, and thus its variables have very little changes over time.

**Figure 7:**
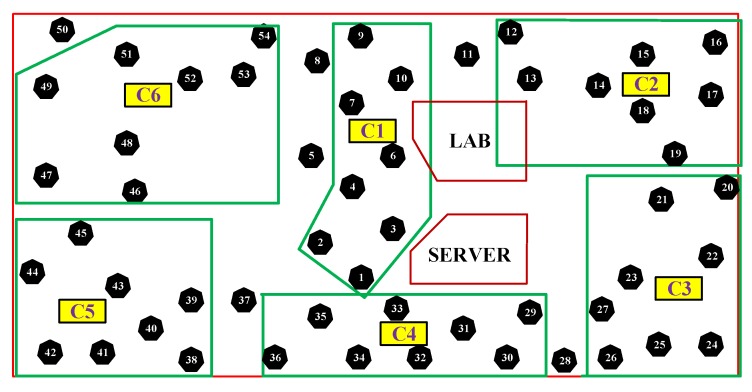
The cluster structure of deployed sensors [57].

Our proposed EDC technique has been implemented in six cluster-based structures, namely C1–C6. Each cluster consists of eight sensor nodes, and thereby a total of 48 out of 54 sensors are chosen to evaluate the performance of the proposed technique. Figure 7 shows the locations of six clusters and the deployed sensor nodes of each cluster as well as the excluded sensors, which are out of the clusters’ range. For simplicity, we assume there is a single CH in each cluster, and the rest of the nodes are the sensor nodes connected to CH.

In our work, the simulations on IBRL dataset have been carried out on only the temperature data from 28 February to 8 March (10 days) collected by 48 sensor nodes. The collected data are in a matrix form at the CHs and all CHs contain a total of 1,382,400 measurements where each CH has 230,400 measurements. The missing values and invalid readings in the observations are replaced by the median value of the collected observations during data preprocessing stage for the continuity. To be realistic, measurements of each CH are segmented into 240 segments or time-interval where an individual segment has 960 measurements. The measurements of each segment are used as an input to our proposed algorithms to evaluate the performance of the proposed technique for each time-interval. Two different rules, namely square-root and Sturge’s, have been considered to specify the number of partitions or clusters for input measurements.

Apart from this, the temperature data in the real world at two consecutive time points have a high degree of similarity, as mentioned in [64]. This type of correlation is normally referred to as temporal similarity or correlation. The calculation of the reading difference of any two consecutive time points of three different sensors is highlighted [64]. The results show only 10% reading difference is greater than 0.1. Hence, strong temporal similarities could be observed in our simulated data due to the same dataset is considered for the performance evaluation of our proposed EDC technique.

### 5.2. Performance Metrics

The performance evaluation of our proposed EDC technique has been accomplished using several performance metrics. The metrics are mainly mean absolute deviation, mean absolute percent error, accuracy, and data reduction percentage. The listed performance metrics are defined as follows:

- Mean absolute deviation (MAD) measures the average deviation of each data point in a data stream from its central value. It describes the variability of a data stream and can be formulated as in Equation (20):
  (20) $$MAD=\frac{1}{\left(m\times T\right)}\sum_{i=0}^{m}\sum_{t=1}^{T}|X\left(i,t\right)-\mu \left(i,T\right)|,$$
  where $X\left(i,t\right)$ is a discrete data point for a specific sensor node and $\mu \left(i,T\right)$ is a central value of the whole data stream in a given period of that particular sensor node.
- Mean absolute percent error (MAPE) measures the mean absolute percentage deviation between each data point and the central value, which can be expressed as in Equation (21):
  (21) $$MAPE=\left\{\frac{1}{\left(m\times T\right)}\sum_{i=0}^{m}\sum_{t=0}^{T}\left(|\frac{X\left(i,t\right)-\mu \left(i,T\right)}{X\left(i,t\right)}|\right)\right\}\times 100.$$
- Accuracy measures the retained originality of the real data after applying our proposed technique for data reduction. It is calculated by subtracting the mean absolute percentage error of a given dataset from the 100 percent and is defined as in Equation (22):
  (22) $$Accuracy=\left(100-MAPE\right)\%.$$
- Data reduction percentage (DRP) measures the difference between the original data $d_{o}$ and the reduced data $d_{r}$ then the difference is divided by the $d_{o}$ and finally the obtained result is multiplied by 100. The DRP can be computed as in Equation (23):
  (23) $$DRP=\left(\frac{d_{o}-d_{r}}{d_{o}}\right)\times 100.$$

## 6. Results and Analysis

The proposed EDC technique presented in Section 4 has been implemented using the Python environment and simulated with real-world temperature measurement data collected from IBRL. We investigate how much data reduction is achieved and also the accuracy of the proposed technique. Basically, the accuracy describes how much the reduced data retains the fundamental properties of the original data, and it is inversely proportional to the data reduction error. If the reduction error is high, then the accuracy is low and vice-versa. Therefore, the associated errors of the proposed technique were investigated prior to the accuracy calculation. Afterwards, the accuracy of the proposed technique, including three modules (HDC, RODS, and V-RODS) were computed and compared to the most popular conventional techniques named K-means and K-medoids [9,15,46]. These algorithms were mainly used for data clustering and reduction of the time-series data. The K-means and K-medoids have been implemented based on the following basic steps.

1. Declaration of predefined number of clusters K.
2. Initialization of number of K randomly as the initial cluster centroids.
3. Assignment of data points to their closest cluster centroid according to the Euclidean distance function.
4. Computation of the centroids using the mean or median of all data points in each cluster.
5. Repetition of steps 2–4 until the same data points are assigned to each cluster in consecutive rounds.

### 6.1. Impact of the Variation of the Number of Clusters on the In-Network Data Reduction Percentage

**Figure 8:**
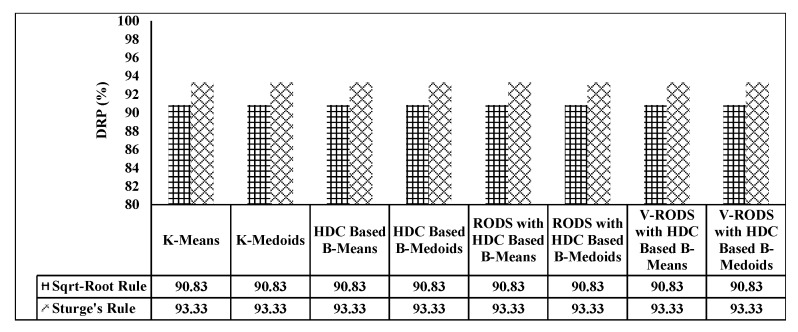
Performance of all evaluated techniques in DRP using two different rules when the K values are set to 11 and 8 accordingly.

In terms of in-network data reduction, a large number of data has been chosen as mentioned earlier, which were used to evaluate the reduction performance of the conventional and proposed techniques. It is presumed that the DRP is highly influenced by the variation of the number of clusters K values. Whereas, the data reduction accuracy is highly affected by regulating the K values in order to maximize the DRP. Thereby, two different K values were set based on two different rules such as square-root (K = 11) and Sturge’s (K = 8) for all evaluated techniques. Therefore, two different data reduction percentages were observed for two different rules. DRP values for all techniques are computed based on Equation (23) and plotted in Figure 8. It can be observed that when the number of clusters decreases, the DRP increases. Hence, the investigation was carried out for the performance of our proposed technique, especially in terms of data reduction accuracy varying two different K values.

### 6.2. Performance Evaluation of the Proposed HDC on Data Reduction Accuracy

**Figure 9:**
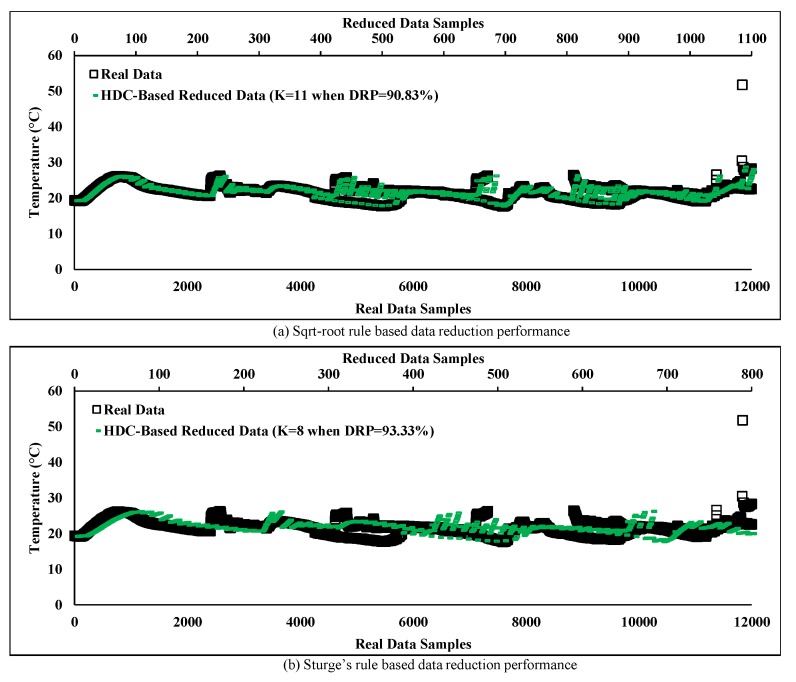
Performance comparison of HDC-based reduced data with real data using two different numbers of clusters. (**a**) The square-root rule (K = 11 when DRP = 90.83%); (**b**) Sturge’s rule (K = 8 when DRP = 93.33%).

In this section, the HDC technique has been evaluated based on different criteria such as DRP, MAD and accuracy in order to provide in-network data reduction. The cluster C1 consisting of eight sensor nodes has been taken into account for identifying the factors that affect the data reduction accuracy of the proposed technique. Impact of the number of clusters on data reduction performance has been illustrated in Figure 9, where the variation of the temperatures is highlighted over time for original and HDC-based reduced data. It can be observed that the number of partitions or clusters has a great influence on performance. For simplicity, we have chosen 100 (from 1 to 100) segments equivalent to 12,000 real data points to evaluate the performance when the number of clusters K is set to 11 and 8 according to the square-root and Sturge’s rules, respectively, for each segment. In the HDC technique, the real number of data (12,000) is reduced to 1100 and 800, respectively, which are plotted at the top horizontal lines in Figure 9a,b. It can be observed that when the number of clusters decreases, the deviation of the reduced data increases along with the DRP. From Figure 9a, it can be clearly seen that the reduced data maintains a trend similar to the real data when the number of clusters is set to 11. On the other hand, when it is set to 8 (in order to increase the DRP further), the reduced data curve deviates from the real data curve as presented in Figure 9b. Hence, it can be concluded that the number of clusters is inversely proportional to the MAD and DRP, thus determining the number of clusters is an important factor for reducing in-network redundant data.

**Figure 10:**
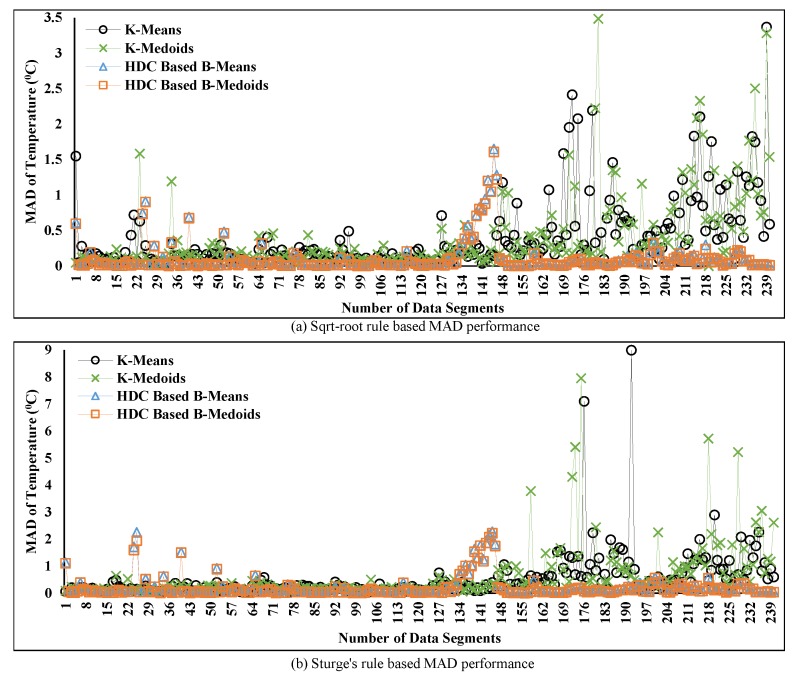
Performance comparison of proposed HDC-based B-means and B-medoids with conventional K-means and K-medoids versus the number of data segments. (**a**) Mean absolute deviation (MAD) versus square-root rule (K = 11); (**b**) MAD versus Sturge’s rule (K = 8).

In Figure 10, the obtained MAD represents the data reduction error for the proposed and conventional techniques, which has been investigated using two different K values. The obtained MAD comparison between the proposed HDC-based and conventional technique is depicted in Figure 10. The K-means and K-medoids are considered as two prototypes of the conventional technique, and the HDC-based B-means and B-medoids are considered as two prototypes of the proposed technique. Moreover, the variation of the obtained MAD is shown in the vertical lines, which changes over time, and the number of data segments or period is represented in the horizontal lines. From the plotted data in Figure 10, it can be observed that the MAD changes over time for both techniques due to two main factors including determining the number of clusters K values for a segment and the temporal correlation among data within a cluster. For example, when the K value is larger and temporal data correlation of a cluster is higher, the associated MAD is lesser for both techniques and vice-versa. However, the obtained MAD from K-means and K-medoids is mostly higher than the HDC-based B-means and B-medoids in the case of both rules or K values in Figure 10a,b because the temporal correlation among data of each cluster is less. Apart from this, although our proposed technique obtains less MAD and outperforms the conventional techniques, it exceeds the standard maximum error threshold, i.e., $T_{e}=$ ±0.5 °C where subscript *e* indicates “error”. This threshold value has been reported in [5,28] for environmental temperature data. The investigation on the minimization of the exceeded MAD has been covered in the subsequent analysis.

**Figure 11:**
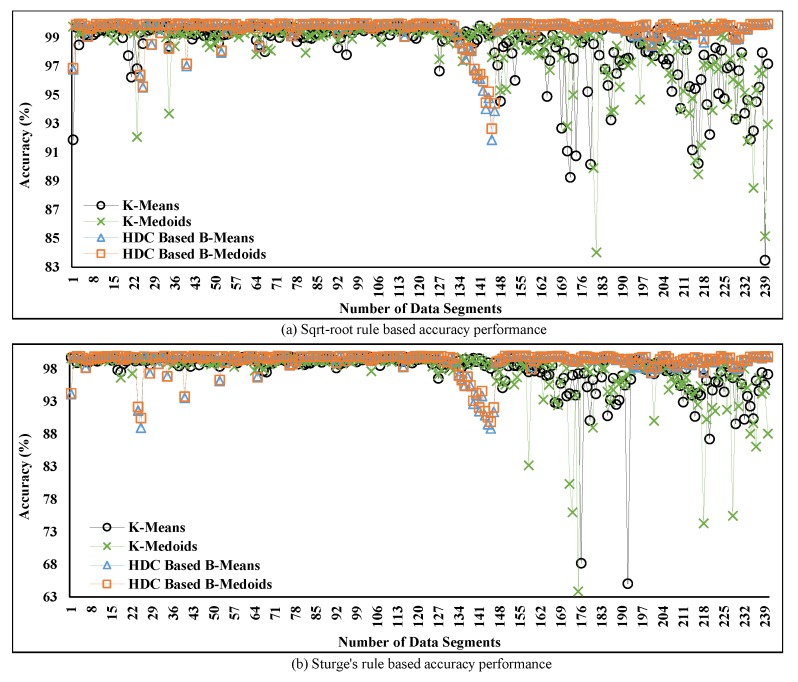
Performance comparison of proposed B-means and B-medoids with conventional K-means and K-medoids versus the number of data segments. (**a**) Accuracy versus square-root rule (K = 11); (**b**) accuracy versus Sturge’s rule (K = 8).

In Figure 11a,b, the percentage of accuracy over time is presented based on two different K values and is computed from the obtained MAD in Figure 10 using Equations (21) and (22). This computation is carried out due to the inversely proportional relation between accuracy and the obtained MAD as mentioned earlier. Thereby, the final accuracy is computed by subtracting the computed percentage of MAD of each segment from the maximum accuracy of that particular segment. The plotted data show that the accuracy of the proposed technique maintains stable condition over time except for certain periods. The reason for this discrepancy can be the noisy data collection at those particular periods. Accuracy is severely influenced by the noisy data collection, which is observed in the cases of both rules from Figure 11a,b.

Indeed, it is claimed that accuracy is influenced not only by the number of K values but also by some other factors, especially in the case of the conventional techniques. In particular, the quality of the collected data can be one of those critical factors that can affect accuracy as well as the MAD. The following sections deal with the outcome of the investigation on the different quality of data collected from different sensor nodes.

### 6.3. Performance Evaluation of Proposed HDC on Data Reduction for Normal Data

In this section, we choose 100 (from 140 to 240) segments of data from “sensor node 9” in C1 for simplicity, where the total number of data samples is 12,000. The data reduction performance is evaluated based on Sturge’s rule (K = 8). The selected samples are assumed as normal real data due to the closeness among their consecutive readings and because the overall data trend maintains stability over time. Afterwards, the proposed technique is applied to 12,000 real data samples, reduced them to 800 data samples and compared the performance of reduced data with normal real data.

**Figure 12:**
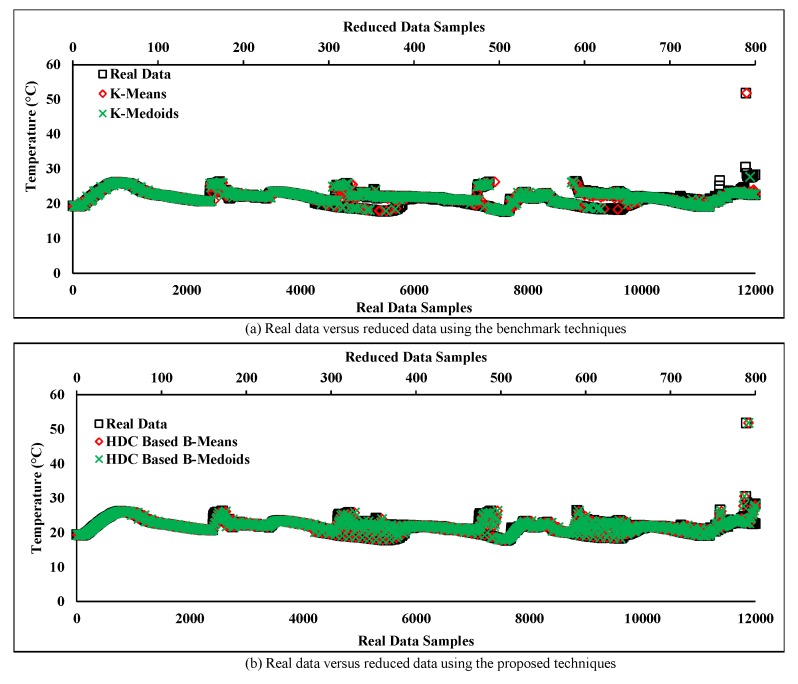
Performance comparison of normal real data with reduced data using the proposed and conventional techniques versus time. (**a**) Temperature versus normal real and reduced data based on conventional techniques; (**b**) temperature versus real and reduced data based on the proposed technique.

Figure 12a,b shows the originality between the real and reduced data pattern after the redundancy reduction using conventional and proposed techniques. The results show that the originality of the reduced data samples is almost equal to the real data samples for both techniques because the deviation of the reduced data of both techniques from the real data is less. Hence, according to the obtained results, it can be claimed that the real data from a normal sensor node has a strong temporal correlation and thus both techniques performed well in terms of accuracy during normal sensor data collection even after a significant amount of data reduction. However, the reduced data by the conventional techniques have slightly deviated from the real normal data at the end for its high sensitivity to data variation.

### 6.4. Performance Evaluation of Proposed HDC on Data Reduction for Noisy Data

**Figure 13:**
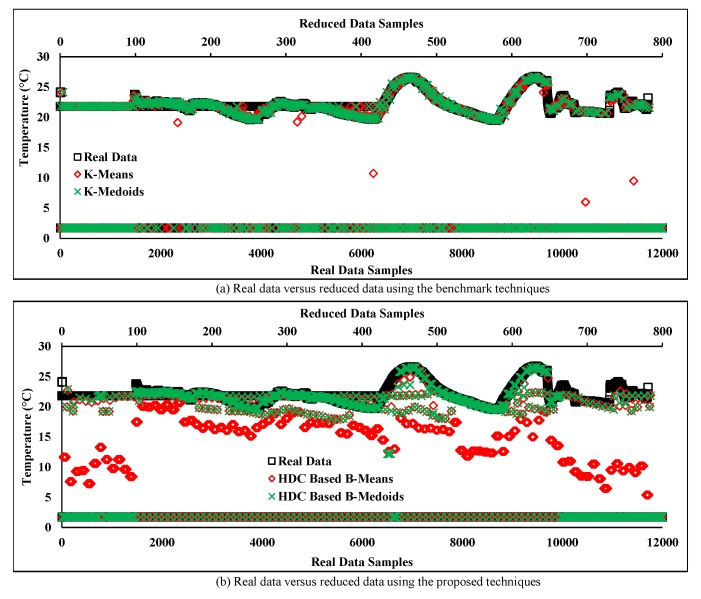
Performance comparison of real noisy data with reduced data using the proposed and conventional techniques versus time. (**a**) Temperature versus noisy real and reduced data based on conventional techniques; (**b**) temperature versus noisy real and reduced data based on the proposed technique.

In this section, the same number of data samples are taken at the same time period as Section 6.3, but from a different sensor node (“sensor node 6”) of the C1. This node is considered as a faulty sensor node as the sensor readings are changing frequently over time. Figure 13 depicts the real data stream associated with faulty sensor readings where the normal readings are assumed less than the faulty sensor readings in a particular period. However, in the normal real data, the temperature range in between 19 and 25 °C is considered as “normal” according to the obtained data from a normal sensor node shown in Figure 12 within the same period. Apart from this, some collected data are far away from the normal data (i.e., between 0 and 5 °C) and that are considered as “noisy” sensor data or outliers.

Figure 13a illustrates the comparison of real data with the reduced data obtained from the conventional techniques based on two different prototypes. The results show that some of the data samples obtained from K-means have shifted away from the real data after reducing redundant data. Moreover, the reduced data attained from the K-medoids prototype have not deviated much from the normal readings but it is maintaining both data pattern (normal and faulty) rigorously over time. Thereby, the overall pattern of the original data may be changed and degraded the accuracy by introducing large MAD.

Figure 13b depicts the results of the HDC-based proposed technique and compares the obtained results with the real data. The plot shows most of the faulty data samples has deviated from the noisy data pattern to the normal data pattern but they are still away from the normal data trend in the cases of both prototypes. As a result, the accuracy may decrease of the proposed technique while collecting noisy data from faulty sensor node.

Hence, the data collection from faulty sensor nodes is considered as another critical factor, and will be investigated in this paper. The investigation will be carried out on the noisy data to improve the overall accuracy with the minimum deviation errors in the subsequent sections.

### 6.5. Performance Evaluation of the Proposed Error-Aware Technique (RODS-HDC) on Data Reduction Accuracy

This section discusses the performance of error-bound guaranteed proposed RODS with HDC-based technique based on two different rules. According to Equation (14), the data stream was classified into noisy and normal by the range of the particular stream. The width of each cluster depends on the range of a particular data stream. Thereby, the width value of a cluster has been set to 1 as a threshold point. When the cluster-width becomes larger than the predefined threshold, the data stream will be counted as noisy and vice-versa. Afterwards, outlier detection will take place from the selected noisy data stream. Hence, the detected outliers will be replaced by the computed median value of that particular noisy data stream. In order to reduce the cluster-width, this detection and replacement phase continues until the value of cluster-width becomes less than or equal to the threshold point. Thus, the data of each cluster deviates from the central value to maximum $\pm 0.5\;{}^{\circ }C$ of that particular cluster. Therefore, the cluster width has been considered as an important factor as it can influence the MAD and accuracy of the proposed technique.

**Figure 14:**
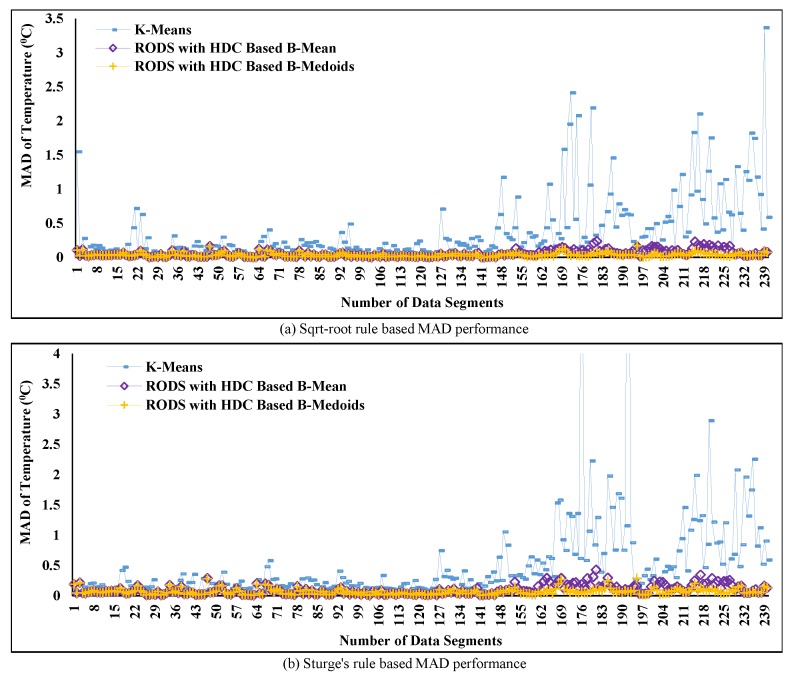
Performance comparison of proposed RODS with HDC-based B-means and B-medoids with conventional K-means versus the number of data segments over time. (**a**) MAD versus square-root rule (K = 11); (**b**) MAD versus Sturge’s rule (K = 8).

Figure 14 shows the performance of the proposed RODS with HDC using the square-root and Sturge’s rules for all segments of C1 by defining the maximum average deviation error $\pm 0.5\;{}^{\circ }C$. The results in Figure 14a highlight the MAD of our proposed RODS with HDC when K value is set to 11. From the plot, it can be clearly observed that the MAD was maintained below the predefined maximum deviation error due to outliers consideration. Therefore, it can be claimed that the error-bound guaranteed RODS with HDC technique performs better than the without error-bound guaranteed conventional techniques as well as HDC-based proposed technique.

However, the RODS with HDC-based B-means prototype generates a slight higher deviation error than the B-medoids during a certain period, but it still remains within the predefined threshold value. This increment of MAD (B-means) can be occurred by the remaining outliers because the mean value is highly affected by the outliers [58].

Figure 14b presents the performance of the proposed error-bounded technique, where the number of clusters was 8, i.e., K = 8 for increasing the percentage of data reduction. Afterwards, the obtained MAD using both RODS with HDC-based prototypes was investigated and compared with the conventional techniques. From Figure 14b, it can be seen that the generated MAD is slightly higher for both prototypes than the obtained MAD in Figure 14a over time but still lower than the predefined threshold error.

**Figure 15:**
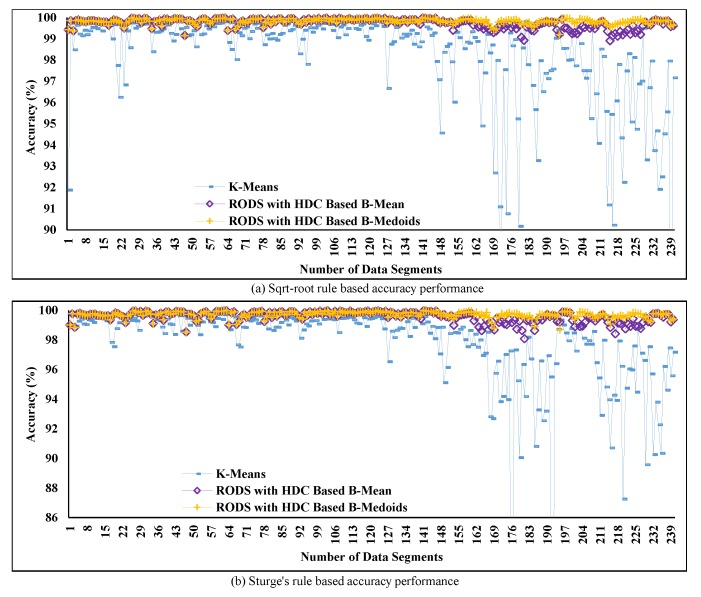
Performance comparison of proposed RODS with HDC-based B-means and B-medoids with conventional K-means versus the number of data segments over time. (**a**) Accuracy versus square-root rule (K = 11); (**b**) accuracy versus Sturge’s rule (K = 8).

On the other hand, the accuracy of the proposed RODS with HDC-based technique was compared with the conventional techniques and shown in Figure 15a,b. The outcomes show that the obtained accuracy from the proposed technique is significantly improved compared to conventional technique and retained the almost similar outcomes over time by varying the K values between 11 and 8. This improved of the proposed technique in accuracy has retained above 99% originality of the C1 dataset even after reducing a significant amount of the data.

### 6.6. Performance Evaluation of Proposed RODS with HDC on Data Reduction for Normal and Noisy Data

This section highlights the data reduction performance between the original and reduced data using the proposed RODS with HDC-based technique for the normal and noisy sensor data. The selected data streams for these two types of data and other selected parameters are kept the same as Section 6.3 and Section 6.4. Followed by, the performance of both prototypes of the proposed technique was evaluated and compared with the original real-world data.

**Figure 16:**
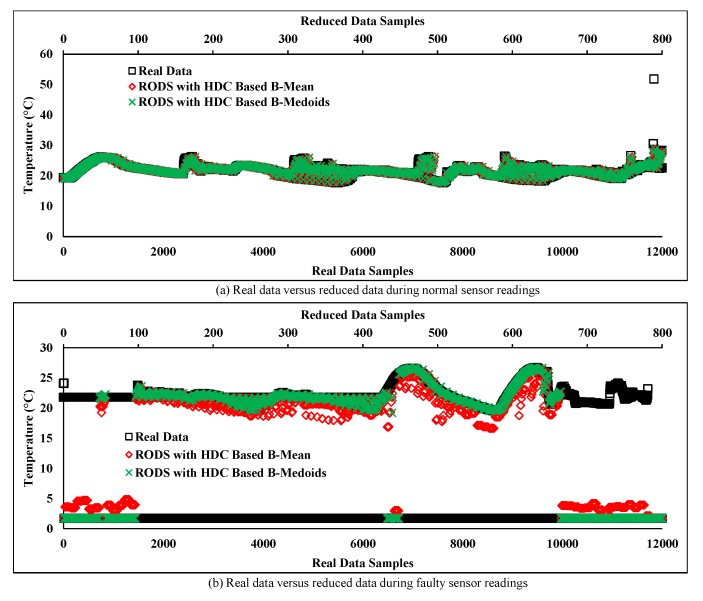
Performance comparison of real normal and noisy data with reduced data using the proposed RODS with HDC-based technique versus time. (**a**) Temperature versus real normal and reduced data based on the proposed RODS with HDC-based technique; (**b**) temperature versus real noisy and reduced data based on the proposed RODS with HDC-based technique.

In Figure 16a, a normal data stream was evaluated where most of the consecutive data points were close to each other except the one residing at the right corner of the plot. This data point can be defined as a random outlier because of the sudden change, which is away from the original data trend. However, our error-bound guaranteed RODS with HDC-based technique successfully detected that outlier and replaced it with the normal data point. This outlier was unable to be detected by the conventional as well as our HDC-based proposed techniques shown in Figure 12a,b, respectively. As a result, the retained originality of the error-bound guaranteed technique has improved by handling the outliers and outperformed the existing techniques.

In contrast, the reduced data stream obtained from the proposed RODS with HDC-based technique as depicted in Figure 16b, where the originality between the proposed RODS with HDC and real data was compared. The plot shows that severe outliers exist in the real noisy data stream where the temperature was recorded between 0 and 5, which are considered as outliers. Therefore, most of the random outliers were detected and replaced with the normal data by both the prototypes of our proposed technique.

However, two more uncovered factors are yet to be addressed. The first one is the reduced data of the RODS with HDC-based B-means prototype, which deviated from the normal data pattern of the real data. This deviation proves that the reduced data stream of the mean prototype is still affected by the outliers. Secondly, both the prototypes of the proposed RODS with HDC-based technique detect false outliers that can be discovered from Figure 16b, where reduced data samples of the proposed technique are still maintaining the noisy data trend within the certain periods. In this case, the proposed technique considers normal data as outliers and replaces them with the actual outliers. As a result, the real data pattern shifted towards the outliers data pattern. Thereby, the real noisy data stream was analyzed and observed where the number of outliers data points within a certain period were found even higher than the normal data points. These types of outliers can be defined as frequent outliers that are generally handled by both the temporal and spatial knowledge of the particular data stream.

### 6.7. Performance Evaluation of Proposed V-RODS with HDC on Data Reduction for Noisy Data

**Figure 17:**
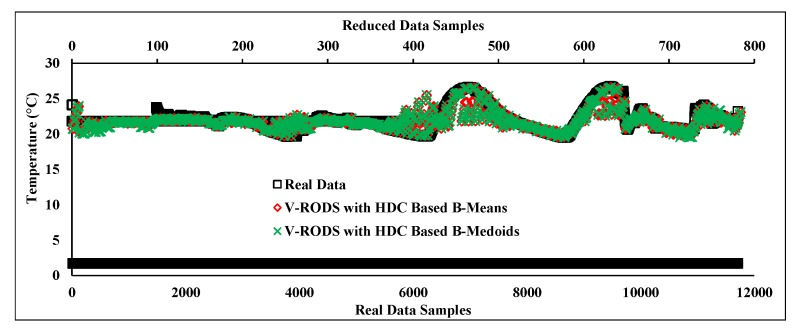
Performance comparison of real noisy data with reduced data using the proposed V-RODS with HDC-based technique versus time.

This section discusses the performance of proposed V-RODS with HDC-based technique during severe noisy data collection that was extracted from a faulty “sensor node 6”. Figure 17 depicts the results of the proposed technique and shows a comparison between the original and reduced noisy data stream. It can be observed that both prototypes of the proposed technique maintain an almost similar trend as compared to the actual trend of the original data stream. This is due to the utilization of both temporal and spatial knowledge of the data stream while detecting and handling outliers (random and frequent) effectively. Thereby, no outlier was retained in the data stream that might deviate the reduced data trend from the actual trend. Hence, the proposed V-RODS with HDC-based technique shows more robustness for detecting and handling both types of outliers.

### 6.8. Impact of the V-RODS with HDC-Based Technique on Data Reduction Accuracy for Noisy Data

This section shows the outcome comparison of the MAD and accuracy between the error-bounded RODS with HDC and the false outliers handling V-RODS with HDC-based technique.

**Figure 18:**
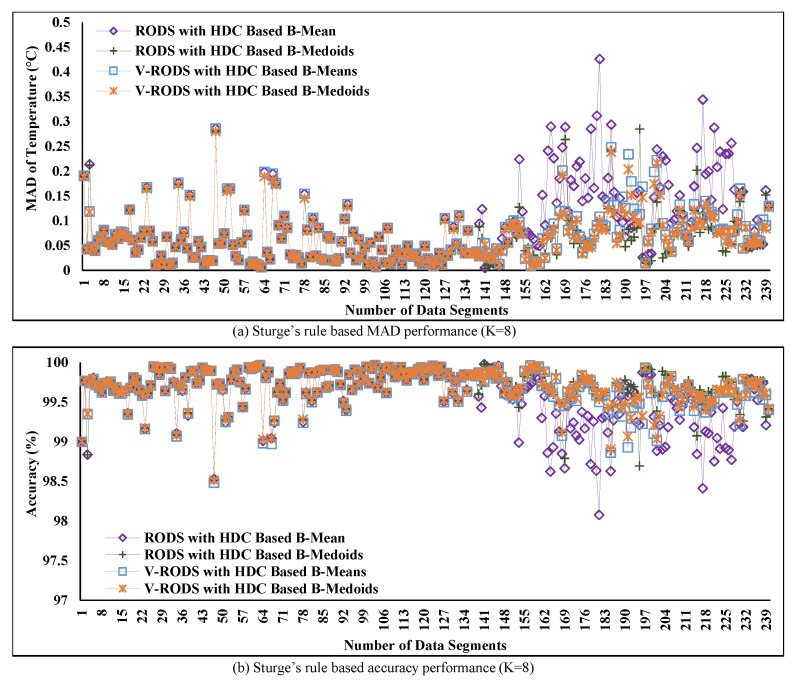
Performance comparison between V-RODS with HDC and RODS with HDC-based techniques versus the number of data segments. (**a**) MAD versus RODS with HDC and V-RODS with HDC-based techniques over time; (**b**) accuracy versus RODS with HDC and V-RODS with HDC-based techniques over time.

The MAD was kept below $\pm 0.30\;{}^{\circ }C$ for both prototypes of V-RODS with HDC that can be seen in Figure 18a. Furthermore, it can be observed that the error-bound guaranteed V-RODS technique outperformed the RODS with HDC technique throughout the period because of handling both types of outliers properly before data clustering. Whereas, only the error-bound guaranteed RODS with HDC-based technique performed well at the beginning. However, it is incompetent at the end while handling the faulty sensor readings, and thus the MAD increased more than $\pm 0.35\;{}^{\circ }C$ for RODS with HDC technique.

According to the obtained MAD of the V-RODS with HDC-based technique, the accuracy was computed and presented in Figure 18b. The plot shows that the accuracy of the V-RODS with HDC-based technique is further improved as compared to another proposed RODS with HDC technique even for the severe noisy data collection from a faulty sensor node.

### 6.9. Impact of the Variation of DRP on MAD after Utilizing the Proposed Technique

**Figure 19:**
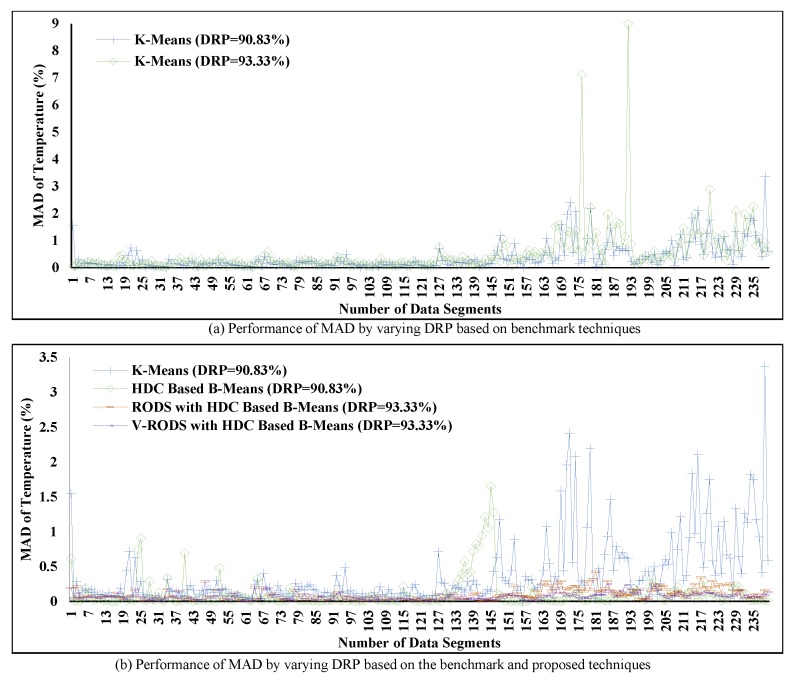
A comparison between the conventional and proposed techniques in terms of MAD and DRP versus the number of data segments. (**a**) MAD versus data reduction percentage (DRP) of the conventional techniques; (**b**) MAD versus DRP of the conventional and the proposed techniques.

This section discusses the changes of MAD with respect to the DRP variation, which can be seen from Figure 19. Figure 19a shows the performance of the existing (K-means) technique for the variation of DRP using two different rules where K = 11 for DRP = 90.83% and K = 8 for DRP = 93.33%. The relation between MAD and DRP can be found as proportional for the existing K-means technique.

However, the error-bound guaranteed proposed RODS with HDC and V-RODS with HDC techniques have proved the simultaneous improvement for both the MAD and DRP while eliminating the redundant data from the data streams. Figure 19b presents the comparison of the benchmark (K-means), the proposed HDC-based (B-means), RODS with HDC-based (B-means) and V-RODS with HDC-based (B-means) techniques. The DRP of benchmark and HDC-based techniques were set to 90.83%, whereas for RODS with HDC and V-RODS with HDC techniques were set to 93.33% to observe the MAD. Thus, it can be clearly seen from the Figure 19b that the RODS with HDC and V-RODS with HDC-based techniques improved MAD along with the DRP significantly. Although the obtained MAD of our proposed RODS with HDC-based technique has a slight increase due to the frequent outliers, it still remains within the predefined threshold and lower than the benchmark and our HDC-based techniques.

### 6.10. Statistical Data Analysis of the Proposed EDC Technique for C1 (Faulty Sensor Node 6)

**Figure 20:**
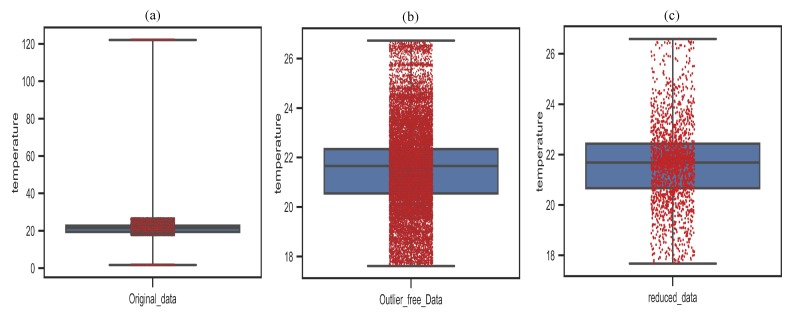
A comparison among original data, outlier-free data by the proposed EDC and the reduced data by the EDC technique versus temperature. (**a**) Original data (1× 28,800); (**b**) outlier-free data (1× 28,800) with the V-RODS algorithm; (**c**) reduced data (1× 1920) with the EDC technique.

The statistical properties of the original, outlier-free and redundancy reduced data are illustrated in this section to evaluate the performance of our proposed technique. Figure 20 shows the statistical properties of the data distribution for different groups of data and the spread of data over the boxplot. In this simulation, 28,560 measurements were collected from the most faulty “sensor node 6” to evaluate the performance of our proposed EDC technique. Five different statistical properties are mainly highlighted in the boxplot: (i) Median is the middle point of the range of data; (ii) lower quartile $\left(Q_{1}\right)$ is a lower percentile of the interquartile range (IQR), which represents the 25th percentile data below the median $\left(Q_{2}\right)$ and displays the potential outliers apart from those below the lower quartile; (iii) upper quartile $\left(Q_{3}\right)$ is an upper percentile of the IQR, which represents the 25th percentile data above from the median $\left(Q_{2}\right)$ and displays the potential outliers that are far from above the upper quartile; (iv) minimum and maximum data values are usually highlighted at below $Q_{1}$ and above $Q_{3}$; (v) the spreading out or distribution of data over time. Figure 20a represents the distribution of the original data where the median value of the distribution is about 22$\;{}^{\circ }C$.

Moreover, it can be seen that the minimum and maximum data of the distribution are far away from $Q_{1}$ and $Q_{3}$, respectively; thereby, these data can be considered as the outliers. However, Figure 20b displays the distribution of the outlier-free data collected by utilizing the V-RODS module of the proposed technique. In this boxplot, it can be observed that the median value of the distribution maintained almost the same as the original dataset. In addition, the minimum and maximum values of the distribution reached close enough to $Q_{1}$ and $Q_{3}$. Thus it can be claimed that the error-bound guaranteed proposed technique maintains outliers accurately and hence there exist no outliers in the distribution. Figure 20c depicts the distribution of the reduced data achieved by utilizing our proposed V-RODS with HDC techniques. The data distribution of this boxplot proved that our proposed technique only removes the redundant data by maintaining almost similar statistical properties as compared to Figure 20a,b.

The average results of all segments collected from C1 (all sensor nodes of C1) are computed based on different statistical parameters for all evaluated techniques as well as real data and represented in Table 2. The results show that the proposed “V-RODS with HDC-based B-means and B-Medoids” techniques performs better than all other evaluated proposed and conventional techniques in terms of all statistical parameters. The main reason is that it detects and smooths both the random and frequent outliers effectively before performing data clustering for data reduction.

### 6.11. Performance Evaluation of the Proposed Technique on the MAD and Accuracy for all CHs (C1–C6)

The performance evaluation of the proposed technique was carried out for all the CHs, which is highlighted in Figure 7. The number of connected sensor nodes at each cluster, number of segments, and other parameters are selected the same as C1. Then, the HDC and RODS with HDC-based proposed techniques were evaluated using two different number of K values in terms of accuracy and DRP. Finally, the obtained results of the proposed technique were compared with the results of the conventional techniques and original dataset.

**Figure 21:**
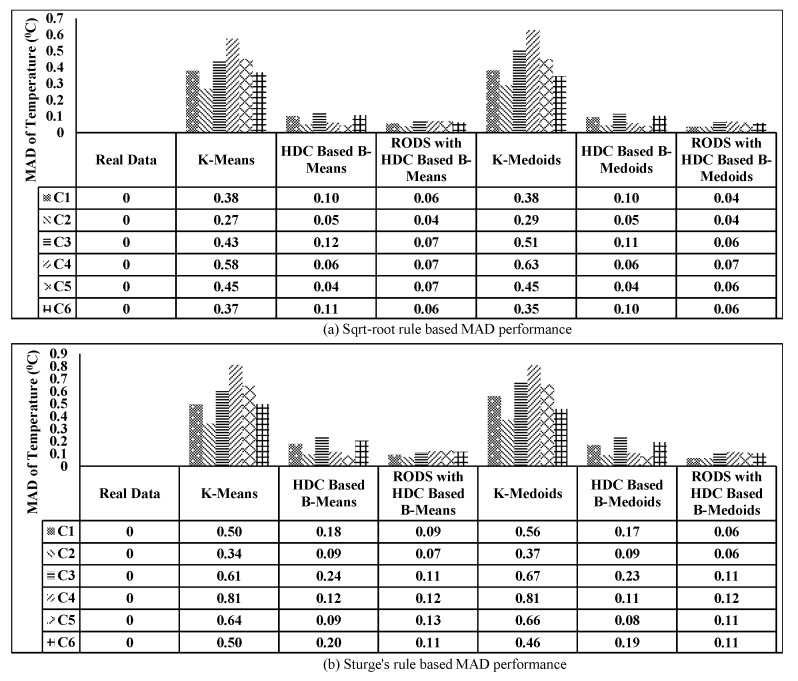
Performance comparison of all evaluated techniques in all selected clusters versus two different rules. (**a**) MAD versus all selected clusters based on the square-root rule; (**b**) MAD versus all selected clusters based on Sturge’s rule.

The mean absolute deviation of the original data was considered 0 as there was no data reduction and error. However, the MAD was introduced when the data reduction was achieved, which consequently increased the DRP. The MAD of all CHs was computed for all selected techniques by averaging of MAD obtained from each CH of all segments and then plotted in Figure 21 based on two different DRPs. Figure 21a shows the MAD in terms of reduction ratio of 10.90:1, where each bar represents an individual CH for each technique. From the plot, it can be clearly seen that both modules of the proposed technique maintained the average MAD to below $\pm 0.2\;{}^{\circ }C$, which is much lower than the evaluated conventional techniques. Furthermore, the significant reduction of MAD in all CHs was observed after applying our error-bound guaranteed module of the proposed technique to below $\pm 0.08\;{}^{\circ }C$.

In Figure 21b, the data reduction ratio was set to 15:1 to observe the associated MAD and it can be seen that the conventional technique performs very poorly as compared to our proposed technique. Whereas, even after increasing the data reduction ratio, the error-bound guaranteed based proposed technique still maintained the MAD below $\pm 0.08\;{}^{\circ }C$.

**Figure 22:**
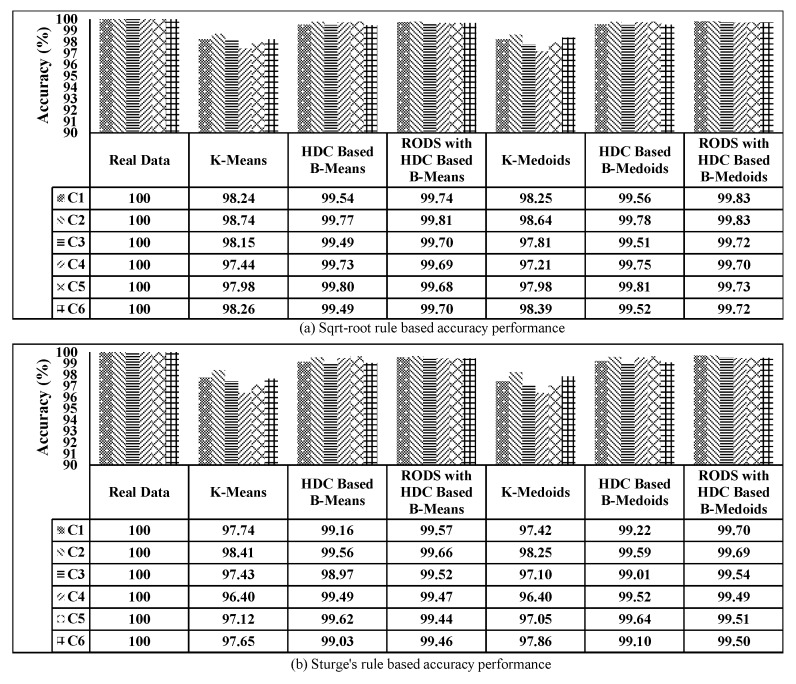
Performance comparison of all evaluated techniques in all selected clusters versus two different rules. (**a**) Accuracy versus all selected clusters based on the square-root rule; (**b**) accuracy versus all selected clusters based on Sturge’s rule.

On the other hand, the obtained accuracy for all techniques has been plotted in Figure 22, which has an inversely proportional relationship with MAD as observed earlier. From the plotted data in Figure 22a,b, it can be further observed that the accuracy of both modules of the proposed technique has improved significantly above 99% compared to the conventional techniques, specifically after utilizing the error-bound guaranteed technique.

The error-bound guaranteed RODS with HDC technique retains more than 99% originality, which is almost equal to the obtained results of our third proposed V-RODS with HDC-based technique. The main reason is the absence of frequent outliers in the rest of the CHs over time.

### 6.12. Complexity Analysis

In this section, two types of complexity, including space and computational, have been analyzed. The estimated space complexity of EDC technique is $O\left(MN\right)$ in the worst case where *M* is the number of sensors, and *N* is the number of data points. In contrast, the evaluated K-means algorithm is required higher than the proposed EDC as it is $O\left(MK+MN\right)$ where K is the number of clusters or randomly initialized centroids for each sensor.

In the proposed technique, the computational complexity has computed in three phases individually. In the first phase, the complexity is required for data clustering, which can be estimated based on Algorithm 1. The cost is $O\left(NK\right)$ where *N* is the number of data points in each cluster, and K is the number of clusters. In the second phase, the RODS is implemented for detecting and smoothing outliers (if any) in the data stream before performing data clustering. The cost for RODS is $O\left(d\right)$ where *d* is the computational cost for detecting outliers and it is d≪NK. Therefore, the overall computational complexity for Algorithm 2 is $O\left(NK+d\right)$ in the worst case, whereas it is $O\left(NK\right)$ for the ideal case. For the third phase, proposed V-RODS computational cost also can be considered as $O\left(d\right)$ for actual outlier detection and replacement. Thereby, the computational complexity of V-RODS Algorithm 3 can be computed for two cases. Case 1, the ideal case cost is $O\left(NK\right)$. Case 2, data clustering while the outlier is detected, validated as an actual or false outlier and repaired them that is considered as the worst case and the cost is $O\left(NK+d\right)$. On the other hand, the required computational time for the benchmark algorithm is $O\left(NKI\right)$ in the best case where *I* is the number of iteration required for convergence.

**Figure 23:**
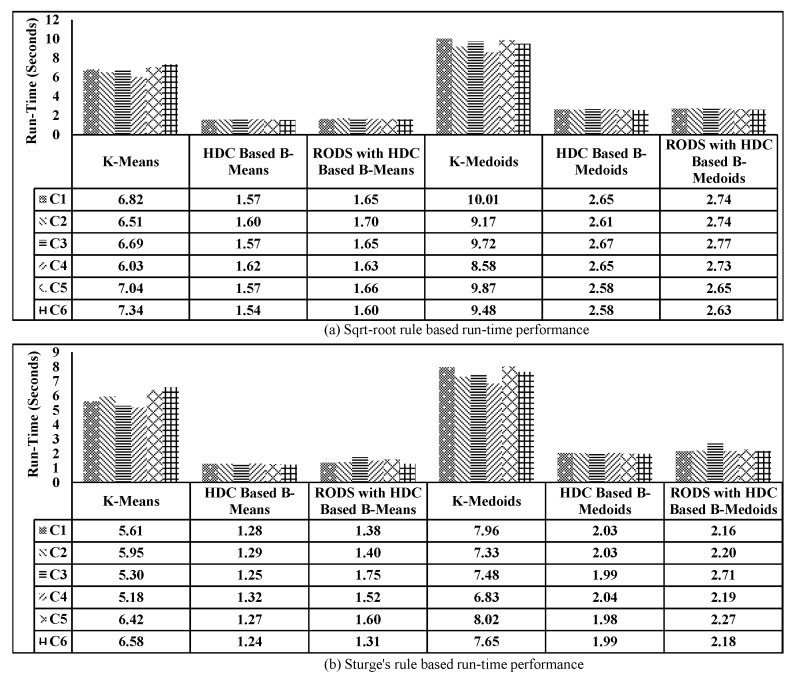
Run-time analysis for square-root and Sturge’s rules.

Figure 23 depicts the average run-time for data clustering at the CHs using two different K values, which was observed in the evaluation of the proposed as well as the benchmark techniques. The specifications of the system are Intel core i7 processor, 3.40 GHz, with 8 GB of RAM where the algorithms were run. From the graph, it can be clearly observed that the proposed EDC technique was largely outperformed the conventional techniques.

### 6.13. Summary

The proposed technique consists of three modules and the main advantage is that the user can select any of the three modules based on the user’s requirement and data quality. The parameters, namely MAD, accuracy and DRP, vary with the changes of the K value. Hence, the selection of the K value can be made based on the desired level of any of these parameters. The main claims of the proposed work are summarized as follows:

Firstly, the investigation on the performance of the HDC-based technique was carried out by varying the number of clusters of the K value. At this stage, the MAD increases when the K value decreases and vice-versa. Based on our investigation, the HDC-based technique performs better than conventional techniques in Figure 10 because there is no random initialization to determine centroids and it is less sensitive to the noisy data.

Secondly, the RODS with the HDC technique has been introduced to provide error-aware data clustering by handling the noisy data. One of the most important benefits of this technique is to provide error-bound guaranteed redundancy reduction by maintaining the MAD within the predefined threshold error even under severe noisy data. Furthermore, in our prototypes (B-means and B-medoids), the MAD values are always maintained below the predefined threshold error as indicated by Figure 14a.

Thirdly, our proposed technique V-RODS with HDC can detect the false outlier and deviation of the reduced data in Figure 16b. This technique detects not only random outliers but also distinguishes frequent outliers by utilizing both the temporal and spatial correlations of the data. Thus, both prototypes of our proposed technique exhibit the similar data pattern as in real data.

Finally, the complexity of our proposed three algorithms is analyzed in two different aspects and compared with the most adopted conventional algorithm [9]. In the first aspect, the proposed three algorithms have the same space complexity of $O\left(MN\right)$, whereas the complexity is $O\left(MK+MN\right)$ for the conventional technique. In another aspect, the computational cost of our proposed technique is $O\left(NK+d\right)$ for the worst case, which is lesser than the best-case computational cost ($O\left(NKI\right)$) of the conventional algorithm.

## 7. Conclusions

Our findings and analysis confirm the fact that data clustering is very useful for reducing in-network data transmission for energy conservation in large-scale periodic WSNs. The EDC was proposed to achieve error-bound guaranteed data clustering for in-network data reduction, hence making it useful for large-scale data generated applications in WSNs.

Our HDC-based technique provides a systematic data clustering to minimize data redundancy. It does not require any random initialization for determining the cluster centroids and it converges the clusters without iteration. One of the most significant contributions of this paper is the development of error-aware data clustering (RODS with HDC) technique. In this technique, the data reduction error (MAD) is maintained below the predefined threshold value by diagnosing the random outliers instead of decreasing the DRP. The implementation of V-RODS with HDC on the other hand contributes to the error-bound guaranteed data clustering for minimizing the in-network redundant data in the case of both random and frequent outliers.

Apart from this, the simulation results of our proposed EDC technique have proven that the data reduction error (MAD) is maintained below the predefined threshold over time. Moreover, it decreases further when the DRP increases. Furthermore, the proposed EDC diagnoses outliers correctly and reduces the data redundancy effectively without affecting the original properties of the data. In addition, the complexity analysis of our proposed EDC shows that both space and computational complexity are lower than the complexity of conventional techniques.

In the future, an extensive performance evaluation of the V-RODS module with HDC will be tested on real-world data that are highly inconsistent, messy and noisy. Recent WSNs usually monitor multivariate data such as temperature, humidity, light and voltage. However, developing a data clustering technique for multivariate data is more challenging than for univariate data. Thus, we intend to conduct further study on multivariate data. Moreover, the energy-efficiency and latency of the proposed EDC technique will be analyzed in the extension of this paper.

## Figures and Tables

**Table 1:** A list of the utilized mathematical notations and their definitions.

Notations	Definitions	Notations	Definitions
$X_{i}$	A specific data stream	*t*	A discrete time point
*T*	Whole period	$\Delta_{d}$	The range of a data stream
*D*	A set of data streams from different sensors	*C*	A set of spatial clusters
$C_{j}$	A spatial data cluster	$SC_{j}$	A temporal data cluster
B	A set of temporal clusters	K	A number of clusters
*W*	Constant width of an individual cluster	$a_{k}$	Individual cluster edge
$\mu_{k}$	Mean value of a cluster	$M_{k}$	Median value of a cluster
$MAD_{k}$	Mean absolute deviation of each cluster	$X^{\prime }$	Aggregated data stream
$d_{i}$	Discrete element of a cluster	$MAD_{B}$	Mean absolute deviation of all clusters
$\xi_{i}\left(l\right)$	A set of standardized scores	$\eta_{i}$	A maximum standard score
${\bar{M}}_{i}$	Median value of a standardized scores	$\bar{{\bar{X}}_{i}}$	A de-standardized data stream
$\Delta_{u}$	Range of updated data stream	${\overset{⌢}{X}}_{m\times n}$	A data matrix
$M_{D}$	Median value of a data matrix	$M_{i}$	Median value of a data stream
${\overset{⇀}{\xi }}_{i}\left(l\right)$	A set of modified standardized scores	${\hat{X}}_{i}$	De-standardized a detected outlier score
$D_{i}$	Absolute deviation between ${\hat{X}}_{i}$ and $M_{D}$	$\Psi_{i}$	A user-defined threshold value

**Table 2:** A comparison table of average values of all segments collected from C1 based on various evaluation metrics when Sturge’s rule was applied for data reduction (K = 8 and DRP = 93.33%).

Techniques	Evaluation Metrics					
	Mean	Median	Std. Dev.	Variance	MAD	Accuracy
**Real Data**	21.54	21.81	4.36	19.01	0	100
**K-Means**	21.52	21.66	3.88	18.08	0.50	97.74
**HDC-Based B-Means**	21.58	21.53	3.53	13.95	0.18	99.16
**RODS with HDC-Based B-Means**	21.61	21.63	3.37	13.77	0.09	99.57
**V-RODS with HDC-Based B-Means**	21.88	21.74	3.13	9.80	0.08	99.67
**K-Medoids**	21.51	21.66	3.87	18.02	0.56	97.42
**HDC-Based B-Medoids**	21.60	21.67	3.74	16.48	0.17	99.22
**RODS with HDC-Based B-Medoids**	21.61	21.69	3.45	14.90	0.06	99.70
**V-RODS with HDC-Based B-Medoids**	21.88	21.75	3.14	9.85	0.06	99.70
